# SUMO Chain-Induced Dimerization Activates RNF4

**DOI:** 10.1016/j.molcel.2014.02.031

**Published:** 2014-03-20

**Authors:** Alejandro Rojas-Fernandez, Anna Plechanovová, Neil Hattersley, Ellis Jaffray, Michael H. Tatham, Ronald T. Hay

**Affiliations:** 1 Medical Research Council Protein Phosphorylation and Ubiquitylation Unit, University of Dundee, Dundee, Scotland DD1 5EH, UK; 2 Centre for Gene Regulation and Expression, College of Life Sciences, University of Dundee, Dundee, Scotland DD1 5EH, UK

## Abstract

Dimeric RING E3 ligases interact with protein substrates and conformationally restrain the ubiquitin-E2-conjugating enzyme thioester complex such that it is primed for catalysis. RNF4 is an E3 ligase containing an N-terminal domain that binds its polySUMO substrates and a C-terminal RING domain responsible for dimerization. To investigate how RNF4 activity is controlled, we increased polySUMO substrate concentration by ablating expression of SUMO protease SENP6. Accumulation of SUMO chains in vivo leads to ubiquitin-mediated proteolysis of RNF4. In vitro we demonstrate that at concentrations equivalent to those found in vivo RNF4 is predominantly monomeric and inactive as an ubiquitin E3 ligase. However, in the presence of SUMO chains, RNF4 is activated by dimerization, leading to both substrate ubiquitylation and autoubiquitylation, responsible for degradation of RNF4. Thus the ubiquitin E3 ligase activity of RNF4 is directly linked to the availability of its polySUMO substrates.

## Highlights

• Characterization of the mechanism of activation of the ubiquitin E3 ligase RNF4
• First indication of E3 ligase activation by SUMO chains

## Graphical Abstract

**Figure:**
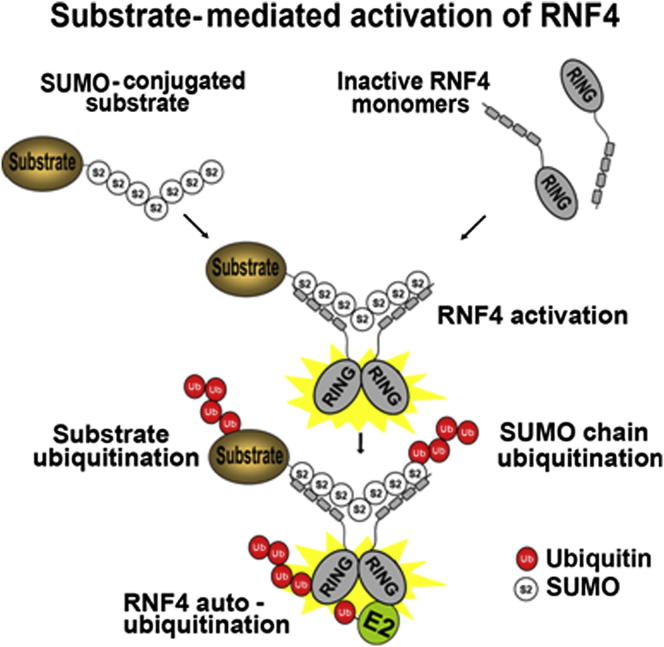

## Teaser

RNF4 is an E3 ligase containing an N-terminal domain that binds its polySUMO substrates and a C-terminal RING domain responsible for dimerization. Rojas-Fernandez et al. show that in the presence of SUMO chains RNF4 is activated by dimerization, leading to both substrate ubiquitylation and autoubiquitylation.

## Introduction

Ubiquitin modification is initiated by the ATP-driven formation of a thioester bond between the C-terminal carboxyl group of ubiquitin and the catalytic cysteine on one of the two E1-activating enzymes, UBA1 or UBA6 (Haas et al., 1982). The activated ubiquitin is then transferred to one of the ∼40 Ubiquitin E2-conjugating enzymes, where it again forms a thioester bond. The E2-Ub complex interacts with ubiquitin E3 ligases that recruit substrates and confer specificity to ubiquitin modification, leading to the formation of either an isopeptide or peptide bond between the ubiquitin C terminus and the ε-amino group of a lysine or the α-amino group of the protein N terminus, respectively (Deshaies and Joazeiro, 2009; Kravtsova-Ivantsiv and Ciechanover, 2012; Scheffner et al., 1995; Tatham et al., 2013). It is thought that there are over 600 E3 ligases encoded in the human genome, and they fall into two distinct groups based on their mechanism. When homologous to E6-AP C terminus (HECT) (Scheffner et al., 1995) and RING-between-RING (RBR) (Wenzel et al., 2011) E3 ligases interact with ubiquitin-loaded E2, the ubiquitin is first transferred onto an active site cysteine residue in the E3, and the resulting thioester bond is attacked by an amino group on the bound substrate to form a peptide bond between ubiquitin and substrate. In contrast, Really Interesting New Gene (RING) E3 ligases function by binding both ubiquitin-loaded E2 and substrate and directly catalyzing transfer of the ubiquitin to substrate. RING E3s prime the ubiquitin-loaded E2 for catalysis by folding the ubiquitin back onto the E2 in a conformation that is optimal for nucleophilic attack by the amino group of the protein substrate (Dou et al., 2012, 2013; Plechanovová et al., 2012; Pruneda et al., 2012).

Small Ubiquitin-like Modifier (SUMO) is encoded by three functional genes in humans. While the conjugated forms of SUMO-2 and SUMO-3 are almost identical and functionally undistinguishable, SUMO-1 is only 45% identical to SUMO-2/3. Like ubiquitin, SUMO-2/3 can form polymeric chains through lysine 11, which is located in a SUMO consensus modification motif (ΨKXD/E, where Ψ is a large hydrophobic amino acid and X is any amino acid). Such a consensus modification motif is lacking in SUMO-1 (Rodriguez et al., 2001; Sampson et al., 2001; Tatham et al., 2001). In humans the SUMO E1-activating enzyme is a heterodimer of SAE1 and SAE2 (Desterro et al., 1999). The SAE2 subunit contains the catalytic cysteine that forms a thioester with the C terminus of SUMO (Desterro et al., 1999). Subsequently, SUMO is transferred from SAE2 to the catalytic cysteine on the unique SUMO E2-conjugating enzyme (UBC9), forming a new thioester (Desterro et al., 1997; Johnson and Blobel, 1997). While UBC9 can SUMOylate substrates directly by recognizing SUMO consensus motifs (Hay, 2005; Rodriguez et al., 2001), its substrate specificity and catalytic activity are enhanced by SUMO E3 ligases (Geiss-Friedlander and Melchior, 2007).

The family of SUMO-Targeted Ubiquitin Ligases (STUbL) functionally link modifications by SUMO and ubiquitin. STUbLs bind to SUMO-modified proteins and induce their ubiquitination (Perry et al., 2008). Recognition of SUMO by STUbLs is mediated by SUMO-interacting motifs (SIM). SIMs were classically defined as a consensus of V/L/I, V/L/I, X, V/L/I or V/L/I, X, V/L/I, and V/L/I, whereas a subgroup of high-affinity SIMs were described as V/I/L/F/Y,V/I,DLT (Hecker et al., 2006; Song et al., 2004; Sun and Hunter, 2012).

RNF4 is a member of the STUbL family and contains four SIMs in tandem at its N terminus which efficiently bind long poly-SUMO2/3 chains (Tatham et al., 2008). The RING domain of RNF4 is located at the very C terminus and is only active as a dimer (Liew et al., 2010; Plechanovová et al., 2011; Plechanovová et al., 2012). RNF4 mediates the ubiquitination and proteasomal degradation of poly-SUMO2/3-modified proteins such as the promyelocytic leukemia protein (PML). The PML protein is a structural component of PML nuclear bodies which are distinct subnuclear structures. In acute promyelocytic leukemia (APL), a chromosomal translocation between the *PML* gene and the gene encoding the retinoic acid receptor alpha (*RARα*) results in the formation of a PML-RARα fusion protein that is responsible for the disease. (Goddard et al., 1991, 1992; Mu et al., 1996). APL can be treated with low doses of arsenic trioxide, which induces polySUMO modification of PML and the PML-RARα fusion proteins which are then ubiquitinated by RNF4 and degraded by the proteasome (Lallemand-Breitenbach et al., 2008; Tatham et al., 2008).

In humans, SUMO proteases counteract the conjugation machinery by depolymerizing SUMO chains and deconjugating SUMO from substrate (Hickey et al., 2012; Melchior et al., 2003). SUMO-specific proteases SENP6 and SENP7 have been shown to act as antagonists of SUMO2/3 chain formation (Mukhopadhyay et al., 2006; Shen et al., 2009), and SENP6 is responsible for the deconjugation of SUMO chains on PML (Hattersley et al., 2011). Thus siRNA-mediated ablation of SENP6 leads to accumulation of SUMO2/3 chains. As SUMO chains are the substrate for RNF4, we used SENP6 depletion as a tool to accumulate SUMO chains in cells to investigate its effect on RNF4 activity. Here, we demonstrate that accumulation of SUMO chains in vivo leads to ubiquitin-mediated degradation of RNF4. In vitro we demonstrate that, at concentrations equivalent to those found in vivo, RNF4 is predominantly monomeric and inactive as a ubiquitin E3 ligase. However, in the presence of SUMO chains, RNF4 is activated by dimerization, leading to both substrate ubiquitination and autoubiquitination, responsible for degradation of RNF4. Thus the ubiquitin E3 ligase activity of RNF4 is directly linked to the availability of its polySUMO substrates.

## Results

### Ablation of SENP6 Expression Leads to the Codepletion of RNF4

**Figure 1:**
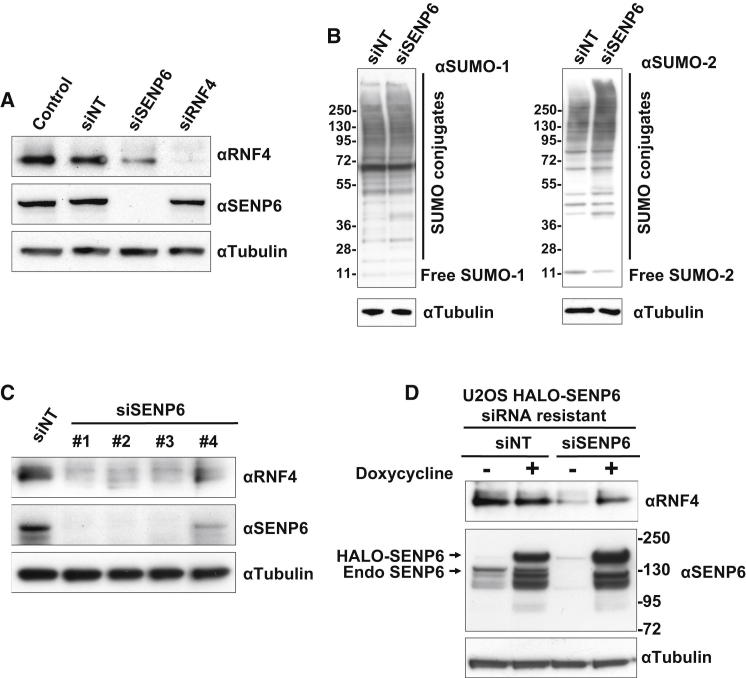
Ablation of SENP6 Reduces RNF4 Levels (A) Untransfected U2OS cells (control) or U2OS cells transfected with nontargeting siRNA (siNT), siSENP6 or siRNF4 were incubated for 72 hr. Protein levels were analyzed by western blotting using antibodies to RNF4, SENP6, and tubulin; also see Figure S1A. (B) U2OS cells were transfected with siNT or siSENP6 and incubated for 72 hr. Levels of SUMO-1 and SUMO-2 conjugates were analyzed by western blotting. (C) U2OS cells were transfected with siNT pool or four individual siRNA oligos directed to SENP6. Western blot analysis was performed as indicated in (A). (D) U2OS Flp T-Rex HALO-SENP6 cells were transfected with siNT or siSENP6 oligo-2 and incubated for 72 hr. Expression of siRNA-resistant HALO-SENP6 was induced with doxycycline 24 hr after transfection and incubated for additional 48 hr. Levels of RNF4, SENP6, and tubulin were analyzed by western blotting.

Under normal growth conditions, cellular levels of SUMO chains are low. However, they accumulate rapidly in response to proteotoxic and genotoxic stress (Golebiowski et al., 2009; Saitoh and Hinchey, 2000; Tatham et al., 2011). These chains can recruit effector molecules such as the SUMO Targeted Ubiquitin Ligase (STUbL) RNF4 or be depolymerized by the chain-specific proteases SENP6 and SENP7 (Shen et al., 2009). siRNA-mediated ablation of SENP6 expression leads to the accumulation of SUMO chains (Hattersley et al., 2011; Mukhopadhyay et al., 2010). As SUMO chains are the substrate for RNF4, it was of interest to determine how substrate availability influenced the activity of RNF4. To accumulate SUMO chains, SENP6 expression in U2OS cells was ablated with siRNA. Under these conditions SENP6 was undetectable by western blotting (Figure 1A), and high-molecular-weight forms of SUMO-1 and SUMO-2/3 accumulated (Figure 1B, and see Figures S1A and S1B available online), but unexpectedly SENP6 ablation led to a dramatic reduction in the levels of RNF4 (Figures 1A and S1A). siRNA directed against RNF4 had no effect on SENP6 expression (Figure 1A). Similar results were obtained in a variety of other human cell lines (Figure S1A). To rule out “off-target” effects, four siRNAs were tested individually. siRNAs 1, 2, and 3 efficiently ablated SENP6 expression and concomitantly reduced RNF4 levels (Figure 1C). In addition, a stable line of U2OS cells was generated containing an siRNA resistant Halo-tagged version of SENP6. Treatment of cells expressing Halo-tagged SENP6 with siRNA to SENP6 led to the ablation of endogenous SENP6 but no reduction in the Halo-tagged SENP6 or RNF4 levels (Figures 1D and S1C). Thus the reduction in RNF4 levels caused by treatment of cells with siRNA to SENP6 is a consequence of SENP6 depletion.

### RNF4 Remaining after SENP6 Depletion Accumulates in SUMO-Containing Nuclear Bodies

**Figure 2:**
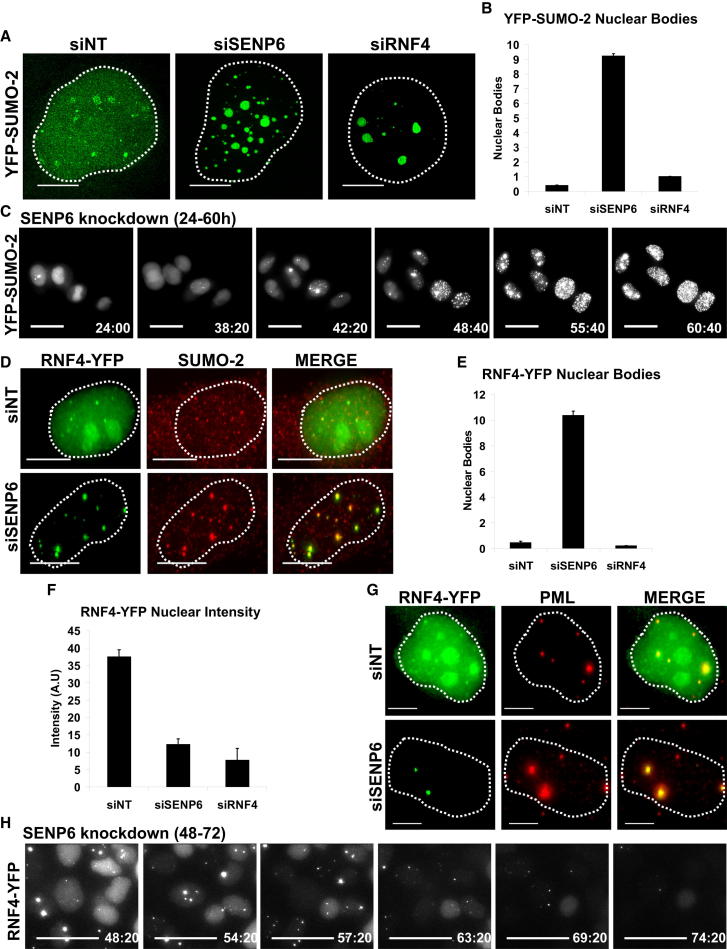
RNF4 Is Relocalized to SUMO-Containing Nuclear Bodies in Response to SENP6 Depletion (A) HeLa YFP-SUMO-2 cells were treated with siNT, siSENP6, or siRNF4. SUMO localization was determined using structured illumination microscopy. Scale bars represent 5 μm. Dashed line indicates location of the nucleus visualized by DAPI staining. (B) HeLa YFP-SUMO-2 cells were transfected in clear-bottom 96-well plates with siNT, siSENP6, or RNF4. Number of YFP-SUMO-2 nuclear bodies was determined by high-content imaging. Data are presented as mean ± SD. (C) HeLa YFP-SUMO-2 cells were transfected with siSENP6 pool. Twenty-four hours after transfection, YFP-SUMO-2 localization was recorded by time-lapse microscopy for 36 hr. Scale bars represent 20 μm. Right-bottom value indicates hours after transfection. See also Movie S1. (D) HeLa RNF4-YFP (green) cells were transfected with siNT or siSENP6 and further stained with a SUMO-2 antibody (red). Scale bars represent 5 μm. See also Figure S2B. (E) HeLa RNF4-YFP cells were transfected with siNT, siSENP6, or siRNF4. The number of RNF4-YFP nuclear bodies was determined by high-content imaging. Data are presented as mean ± SD. (F) High-content quantification of RNF4-YFP nuclear intensity. Data are presented as mean ± SD. (G) HeLa RNF4-YFP (green) cells were transfected with siNT or siSENP6 and further immunostained with a PML antibody (red). Scale bars represent 5 μm. See also Figure S2C. (H) HeLa RNF4-YFP cells were reverse transfected with siSENP6 in clear-bottom microscopy chambers; 48 hr after transfection, RNF4-YFP localization was recorded by time lapse microscopy for 24 hr. Scale bars represent 20 μm. Right-bottom value indicates hours after transfection. See also Movie S2.

To quantify the effect of SENP6 depletion on the levels and subcellular localization of SUMO-2 and RNF4, we employed stable cell lines expressing yellow fluorescent protein (YFP)-tagged SUMO-2 and RNF4. HeLa cells expressing YFP-SUMO-2 were treated with a nontargeting (NT) siRNA or siRNAs directed against SENP6 and imaged by fluorescence microscopy, and quantified by high-content imaging (Figure S2A). Compared to NT siRNA treatment, the number of YFP-SUMO-2-containing bodies is much higher when cells are treated with siRNAs to SENP6 or RNF4 (Figures 2A, 2B, and S2A). To establish the genesis of the YFP-SUMO-2 foci, cells were treated with siRNA to SENP6 for 24 hr and followed for a further 36 hr by time-lapse imaging (Figure 2C; Movie S1).

To determine the fate of RNF4 in cells depleted of SENP6, a stable cell line expressing RNF4-YFP (Yin et al., 2012) was treated with a NT siRNA or siRNAs to SENP6, and RNF4-YFP fluorescence was analyzed. SENP6 siRNA treatment results in a significantly higher number of RNF4-YFP-containing bodies than the NT siRNA control (Figures 2D, 2E, and S2B). Consistent with our western blot studies (Figure 1A), nuclear RNF4-YFP fluorescence is reduced in SENP6 siRNA treated cells (Figures 2D and 2F). Costaining of SENP6-depleted RNF4-YFP-expressing cells with an antibody to PML indicated that nuclear foci in which RNF4-YFP accumulated were PML bodies (Figures 2G and S2C). To establish the time course of RNF4 depletion when SENP6 is depleted, RNF4-YFP-expressing cells were treated with siRNA to SENP6 and YFP fluorescence monitored by time-lapse imaging. This shows RNF4-YFP initially accumulating in nuclear bodies and then disappearing completely (Figure 2H; Movie S2). Thus siRNA-mediated ablation of SENP6 expression leads to the depletion of RNF4 from the nucleoplasm and its relocalization to PML bodies where SUMO-2/3 accumulates.

### In the Absence of SENP6, SUMO Chains Induce the Autoubiquitination and Proteasomal Degradation of RNF4

**Figure 3:**
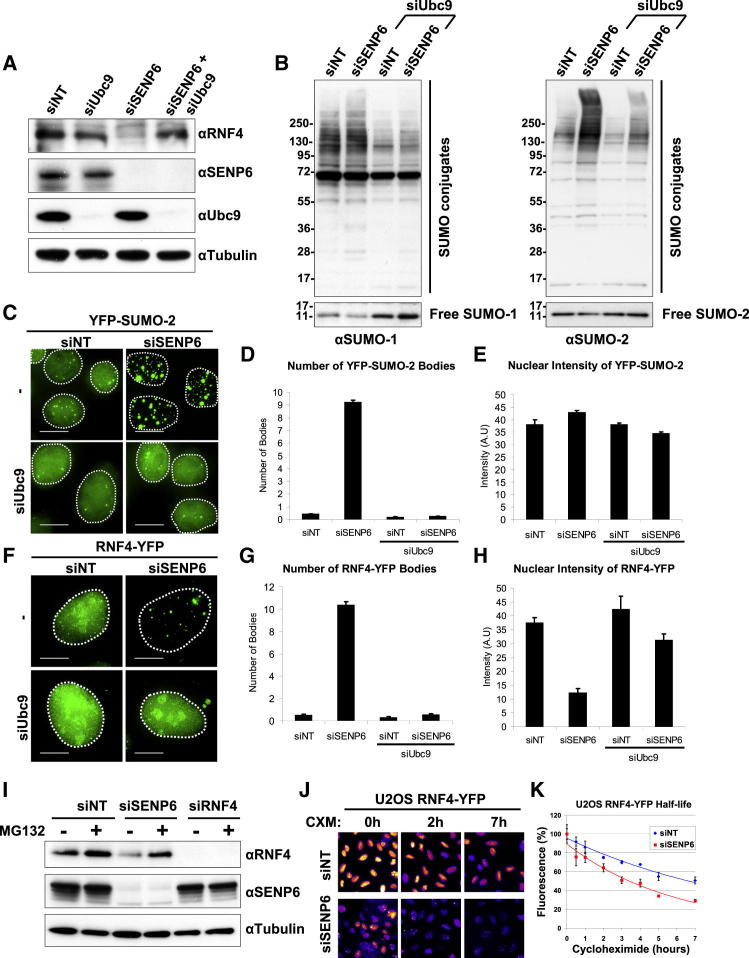
Inhibition of SUMO Conjugation Rescues Levels of RNF4 in SENP6-Depleted Cells (A) U2OS cells were transfected with siNT, siUbc9, siSENP6, or a combination of siSENP6 plus siUbc9. Levels of RNF4, SENP6, Ubc9, and tubulin were analyzed by western blotting. (B) U2OS cells were transfected with siNT or siSENP6 or combinations of siUbc9 and siNT or siUbc9 and siSENP6. Levels of SUMO-1 and SUMO-2 conjugates were analyzed by western blotting (lower panels show longer exposure of free SUMO-1 and SUMO-2, respectively). (C) HeLa YFP-SUMO-2 cells were transfected with siNT or siSENP6 or combinations of siUbc9 and siNT or siUbc9 and siSENP6. Scale bars represent 5 μm. Dashed line indicates the nuclear localization determined by DAPI staining. (D and E) Alternatively, HeLa YFP-SUMO-2 cells were transfected with siNT or siSENP6 or combinations of siUbc9 and siNT or siUbc9 and siSENP6. Cells were analyzed by high-content imaging microscopy, and the number of YFP-SUMO-2 nuclear bodies (D) or YFP-SUMO-2 nuclear intensity (E) was determined. Data are presented as mean ± SD. (F–H) (F) U2OS RNF4-YFP cells were transfected with siNT or siSENP6 or combinations of siUbc9 and siNT or siUbc9 and siSENP6. Scale bars represent 5 μm. Dashed line indicates the nuclear localization determined by DAPI staining. Alternatively, HeLa RNF4-YFP cells were transfected with siNT or siSENP6 or combinations of siUbc9 and siNT or siUbc9 and siSENP6. Cells were analyzed by high-content microscopy, and the number of RNF4-YFP nuclear bodies (G) or RNF4-YFP nuclear intensity (H) was determined. Data are shown as mean ± SD. (I) U2OS cells were transfected with siNT, siSENP6, or siRNF4 for 48 hr and then incubated for an additional 4 hr with DMSO or 25 μM MG132. RNF4, SENP6, and tubulin levels were analyzed by western blotting. (J) High-content determination of RNF4 turnover. Cycloheximide was added to U2OS cells expressing hRNF4-YFP that had previously been transfected with either siNT or siSENP6 for 48 hr. High-content images are shown in an intensity scale (white/yellow, high intensity; blue/black, low intensity). (K) Quantitation of the data shown in (J); n = 8, data are represented as mean ± SD.

SENP6 depletion leads to accumulation of SUMO in PML bodies and a concomitant decrease in the levels of RNF4. To determine if the accumulation of SUMO induced by SENP6 depletion was causally linked to the depletion of RNF4, SENP6, and the SUMO E2, Ubc9, were codepleted. Treatment of cells with an NT siRNA or siRNA to Ubc9 had no effect on RNF4 levels, whereas treatment of cells with an siRNA to SENP6 reduced the level of RNF4. However, codepletion of Ubc9 and SENP6 restored RNF4 to untreated levels (Figure 3A). Analysis of SUMO-1 and SUMO-2/3 conjugates by western blotting indicated that ablation of Ubc9 expression reduced the level of SUMO conjugates irrespective of codepletion of SENP6 (Figure 3B). To establish the effects of SENP6 and Ubc9 depletion on the subcellular localization of SUMO-2 and RNF4, cells expressing YFP-SUMO-2 or RNF4-YFP were treated with siRNAs to SENP6 or Ubc9, or SENP6 and Ubc9 combined and the cellular YFP fluorescence determined. The subcellular distribution of YFP-SUMO-2 was determined by fluorescence microscopy (Figure 3C), while fluorescence intensity and the number of YFP-SUMO-2-containing nuclear bodies was determined by high-content imaging (Figures 3D and 3E). The number of SUMO-containing nuclear bodies increased when SENP6 expression was ablated, and this was abrogated by codepletion of Ubc9 (Figures 3C and 3D). In contrast, neither depletion of SENP6, Ubc9 nor codepletion altered the total amount of YFP-SUMO fluorescence (Figure 3E). Analysis of the RNF4-YFP fluorescence indicated that SENP6 ablation resulted in a large increase in the number of nuclear bodies containing RNF4-YFP, and this increase was abrogated by codepletion of Ubc9 (Figures 3F and 3G). Total intensity of RNF4-YFP fluorescence was decreased when SENP6 was depleted, but this decrease was abrogated when Ubc9 was codepleted (Figures 3F and 3H). Ubc9 depletion alone had no effect on the levels of RNF4-YFP (Figures 3F and 3H). Thus it appears that the decrease in RNF4 levels observed after SENP6 depletion is a consequence of an increase in the levels of polySUMO. A possible mechanism to explain this observation is that as SUMO chains are the substrate of RNF4, then their accumulation might activate the E3 ligase activity of RNF4, leading to its autoubiquitination and proteasomal degradation. Thus cells were treated with either a NT siRNA or siRNAs directed against either SENP6 or RNF4 and after 48 hr cells were treated with proteasome inhibitor MG132 for a further 4 hr prior to analysis by western blotting. MG132 restored the decrease in RNF4 level accompanying SENP6 (Figure 3I). To establish that RNF4 has a reduced half-life in the presence of SUMO chains, U2OS cells stably expressing RNF4-YFP were treated with an siRNA to SENP6 or a control NT siRNA and protein synthesis inhibited by cycloheximide. High-content imaging showed that RNF4-YFP had a reduced half-life in the presence of SUMO chains (Figures 3J and 3K). Thus it appears that when SUMO chains accumulate, as a consequence of SENP6 depletion, RNF4 undergoes autoubiquitination and is degraded by the proteasome.

### SUMO Chains Activate RNF4

**Figure 4:**
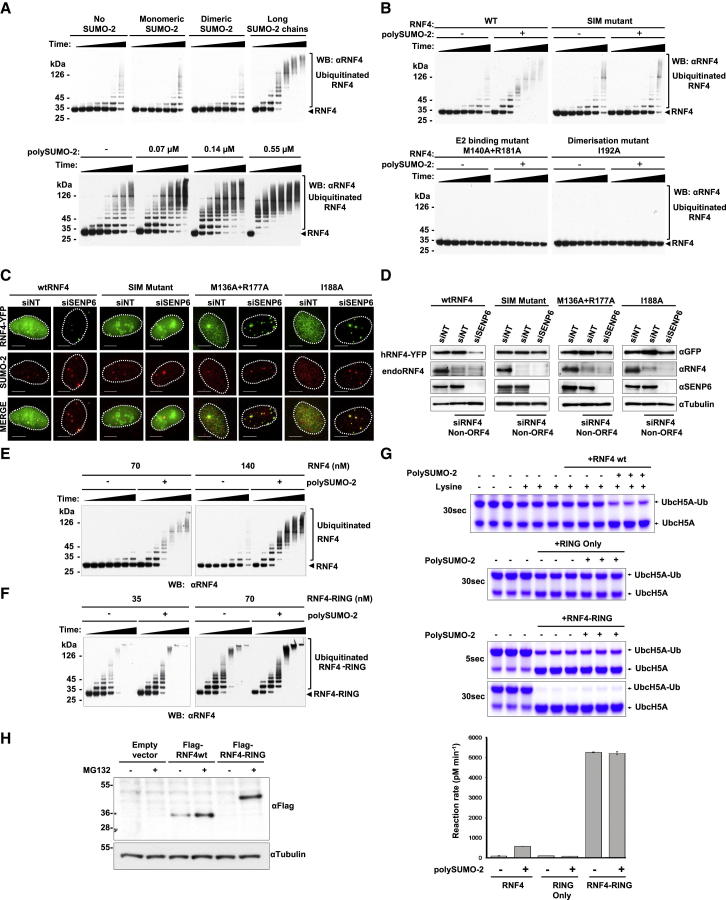
PolySUMO Chains Activate RNF4 In Vitro (A) (Upper panel) RNF4-dependent in vitro ubiquitination reactions were performed either in the absence of SUMO-2 or in the presence of monomeric SUMO-2, dimeric SUMO-2, or long SUMO-2 chains (>5-mers). Samples contained 8.8 μM SUMO-2 (based on monomeric SUMO). Time points were taken at 0, 1, 2, 5, 10, 20, and 40 min. Autoubiquitination of RNF4 was analyzed by western blotting. (Lower panel) SUMO chains activate RNF4 in a concentration-dependent manner. In vitro ubiquitination reactions were performed in the presence of increasing concentrations of long SUMO-2 chains. Concentrations of polySUMO-2 shown indicate concentrations of polymerized SUMO-2 monomer. Time points were taken at 0, 5, 10, 20, 30, 40, and 60 min, and samples were analyzed by western blotting. (B) Autoubiquitination of wild-type RNF4 or RNF4 mutants in the absence or presence of long SUMO-2 chains (2.2 μM). Time points were taken at 0, 1, 2, 5, 10, 20, and 40 min. Samples were analyzed by western blotting with an RNF4 antibody. (C) Stable U2OS cell lines encoding either WT human RNF4 fused to YFP, a SIM mutant of RNF4-YFP, an E2 binding-deficient mutant of RNF4-YFP (M136A, R177A), or a dimerization-defective version of RNF4-YFP (I188A) were transfected with an siRNA oligo targeting only the endogenous RNF4 mRNA (siRNF4 Non-ORF4). After 24 hr incubation, cells were transfected again either with siNT or siSENP6 and incubated for an additional 72 hr. Localization of RNF4-YFP WT and mutants (green) was analyzed together with immunostaining of endogenous SUMO-2 (red). Scale bars represent 20 μm. Dashed line shows the localization of the nucleus determined by DAPI staining. See also Figure S3D. Data are presented as mean ± SD. (D) Western blot analysis of cells treated as in (C), levels of RNF4-YFP, endogenous RNF4, SENP6, and tubulin were determined with specific antibodies. (E) In vitro ubiquitination reactions with the indicated concentrations of wild-type RNF4 were performed either in the absence or the presence of long SUMO-2 chains (2.2 μM). Time points were taken at 0, 1, 2, 5, 10, 20, and 40 min. Reactions were stopped by SDS-PAGE sample buffer, and samples were diluted to the same concentration of RNF4 and analyzed by western blotting. (F) In vitro ubiquitination reactions with the indicated concentrations of RNF4-RING linear fusion were performed either in the absence or presence of long SUMO-2 chains (2.2 μM). Time points were taken at 0, 0.5, 1, 2, 5, 10, and 20 min. (G) Lysine discharge assays. UbcH5A loaded with ubiquitin was mixed with SUMO chains, lysine, and either RNF4, the RING domain of RNF4, or an RNF4-RING fusion. Reactions were terminated after 30 s (or 5 s for the RNF4-RING fusion), fractionated by nonreducing SDS PAGE, and stained with Coomassie blue. Initial rates of reactions were determined (shown as mean ± SD of triplicate reactions) after staining with Sypro Ruby. (H) HeLa cells were stable transfected with either pFires-Puro empty vector, Flag-hRNF4, or a Flag-RNF4-RING fusion. Cells were treated with MG132, and Flag-tagged proteins were detected by western blot using an anti-Flag antibody. Tubulin levels were used as a loading control.

To test the hypothesis that the E3 ligase activity of RNF4 is activated by SUMO chains we undertook in vitro ubiquitination assays (Tatham et al., 2008; Plechanovová et al., 2011; Plechanovová et al., 2012). As a substrate-independent readout of activity, RNF4 autoubiquitination was assessed over time by western blotting with an RNF4 antibody. In the absence of SUMO, RNF4 displays a low level of autoubiquitination activity, and this activity is not appreciably stimulated by addition of monomeric or dimeric SUMO-2. However, the reaction rate and extent of RNF4 autoubiquitination is increased dramatically by the addition of long (>4-mers) SUMO chains (Figure 4A, upper panel, and Figure S3C). Addition of increasing amounts of SUMO chains leads to a dose-dependent increase in RNF4 autoubiquitination (Figure 4A, lower panel). While wild-type RNF4 responds to SUMO chains by increasing autoubiquitination, this response is not observed with a mutant of RNF4 whose ability to bind SUMO has been compromised by mutations in its SUMO interaction motifs (SIMs). Rather, the SIM mutant displays the same low level autoubiquitination in the presence and absence of SUMO chains as that observed for wild-type RNF4 in the absence of SUMO chains (Figure 4B, upper panel). Mutants of RNF4 deficient in E2 binding or dimerization show no autoubiquitination (Figure 4B, lower panel). Thus RNF4 autoubiquitination is activated by its SIM-directed interaction with SUMO chains and requires both E2 binding and RING-mediated dimerization.

An alternative RNF4 functional assay is K63-linked ubiquitin chain synthesis (Tatham et al., 2013). Uev2/Ubc13 generates unanchored K63 chains in the absence of RNF4, and this activity is not influenced by the presence of SUMO chains (Figure S3E, left panel). Addition of RNF4 modestly increased K63 chain synthesis, but in the presence of SUMO chains long K63 polymers accumulate and free ubiquitin is depleted (Figure S3E, right panel). Thus SUMO chains stimulate not just RNF4 autoubiquitination but also the formation of unanchored K63 chains mediated by Uev2/Ubc13.

To establish our findings in vivo, we generated four stable U2OS cell lines encoding wild-type or various mutants of RNF4 fused to YFP: SIM mutant, an E2 binding-deficient mutant (M136A, R177A), or a dimerization defective form (I188A) (Figures 4C, S3A, and S3B). To eliminate endogenous RNF4, an siRNA targeting the non-ORF 5′ UTR of RNF4 (siRNF4 non-ORF4) was used that did not affect expression of RNF4-YFP. Stable cell lines expressing RNF4-YFP WT and mutants were transfected with combinations of siRNF4 non-ORF4, NT siRNA, or siRNA directed against SENP6 and cells stained with a SUMO-2 antibody. While WT RNF4-YFP was degraded, none of the RNF4 mutants was degraded in response to SENP6 depletion. The degradation-resistant E2 binding (M136A, R177A) and dimerization (I188A) mutants formed very large foci associated with SUMO-2 conjugates, while the SIM mutant of RNF4 was not recruited to SUMO foci (Figures 4C and S3D). western blotting confirmed that ablation of SENP6 expression resulted in depletion of WT RNF4-YFP; none of the RNF4 mutants was degraded (Figure 4D). These data indicate that activation of RNF4 required its SIM dependent binding to SUMO chains and the ubiquitin ligase activity of its RING domain in vitro and in cells.

### PolySUMO Chains Induce RNF4 Dimerization

To test the hypothesis that SUMO chains induced dimerization of RNF4, we compared autoubiquitination activity of wild-type RNF4 (Figure 4E) with a version in which full-length RNF4 was fused with the RNF4 RING domain in a single polypeptide (RNF4-RING) (Figure 4F), thus enforcing the dimerization of the two fused RING domains of RNF4. This form of RNF4 was previously shown to be fully active in the ubiquitination of SUMO chains (Plechanovová et al., 2011). At the same concentration of RING domains and in the absence of SUMO chains, RNF4-RING was substantially more active than wild-type RNF4 (Figures 4E and 4F). However, in the presence of SUMO chains the activity of wild-type RNF4 was dramatically stimulated (Figure 4E), whereas little change was observed in the activity of RNF4-RING (Figure 4F). The RNF4-RING fusion in the absence of SUMO-2 chains displayed a comparable level of activity to wild-type RNF4 in the presence of SUMO chains (Figures 4E and 4F). To quantify the effect of SUMO chains on the E3 ligase activity of RNF4, the RNF4-RING fusion and the RING alone, we employed a lysine discharge assay which measures the ability of an E3 ligase to stimulate the transfer of ubiquitin from a ubiquitin-loaded E2 to free lysine. In the absence of SUMO chains WT RNF4 and the RNF4 RING domain only had a similar low level of lysine discharge activity, whereas the RNF4-RING had a 60-fold higher level of activity. Addition of SUMO chains to RNF4 increased the initial rate of lysine discharge by 6.5-fold, but SUMO chains did not alter the activity of the RING alone or the RNF4-RING fusion (Figure 4G). Consistent with these in vitro observations, ectopic expression of RNF4 could be detected by western blotting, whereas an RNF4-RING fusion was almost undetectable under similar conditions. While treatment of cells with proteasome inhibitor MG132 increased RNF4 levels, it had a dramatic stabilizing effect on the RNF4-RING fusion (Figure 4H), suggesting that the RNF4-RING fusion has a faster turnover than RNF4 in cells.

**Figure 5:**
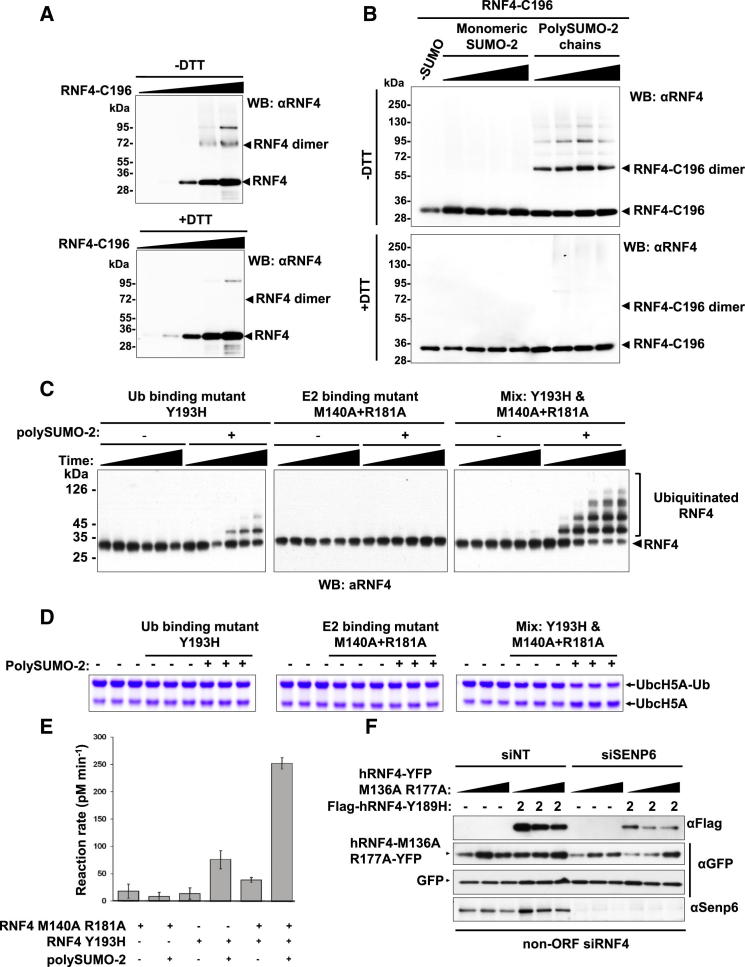
PolySUMO-2 Chains Complement RNF4 Inactive Mutants by Inducing a Functional RING Dimerization (A) Mutant RNF4 C55S, C59S was tagged with glycine-cysteine at its C terminus (RNF4-C196). Increasing concentrations of the bacterial purified protein (0.062, 0.12, 0.25, 0.5, and 1 μM) were incubated at room temperature for 2 hr in the absence of reducing agents. Nonreducing SDS-PAGE was used for detection of disulfide dimer formation (upper panel), and DTT-reduced SDS-PAGE was used to disrupt the disulfide bonds (lower panel). RNF4 was detected by western blotting. (B) A total of 0.1 μM RNF4-C196 was incubated in absence of SUMO as control or with increasing amounts of monomeric SUMO-2 or polySUMO-2 (0.2, 0.4, 1, 2 μM). Disulfide dimer formation was analyzed by nonreducing (upper panel) or reducing (lower panel) SDS-PAGE and western blotting. (C) Autoubiquitination assay of Y193H RNF4, M140A+R181A RNF4 and a mix of the two. Reactions were terminated after 0, 5, 10, 20, 30, or 40 min and analyzed by western blotting. (D) Lysine discharge assay of Y193H RNF4, M140A+R181A RNF4, and a mix of the two. Reactions (in triplicate) were terminated after 30 s and analyzed by Coomassie blue staining. (E) Quantitation of lysine discharge assays was as indicated in Figure 4G. Data are presented as mean ± SD. (F) U2OS cells transfected with non-ORF siRNF4 plus siNT or siSENP6 siRNA were transiently transfected with Flag-hRNF4 Y189H (2 μg) and increasing amounts of hRNF4-YFP M136A, R177A (0.5, 1, and 2 μg) in 12-well plates. Additionally, 50 ng GFP was cotransfected as control. Protein levels were detected by western blotting using Flag, GFP, and SENP6 antibodies.

To directly demonstrate that SUMO-2/3 chains induced RNF4 dimerization, we engineered a cysteine residue into the C terminus of RNF4 that, based on structural predictions, could form a disulphide bond upon RNF4 dimerization. When increasing concentrations of RNF4 C196 were incubated in the absence of reducing agent and the products analyzed by western blotting with an RNF4 antibody, a species accumulated that, based on its molecular mass and its sensitivity to DTT, was consistent with the formation of a dimer (Figure 5A). We therefore incubated RNF4 C196 in the presence of either monomeric SUMO-2 or polySUMO-2/3 and assessed dimer formation as described above. At the RNF4 C196 concentration chosen, very little disulphide linked material is detected in the presence of monoSUMO. However, in the presence of SUMO chains, dimers of RNF4 are rapidly formed (Figure 5B). These data indicate that SUMO chains activate the catalytic activity of RNF4 by inducing its dimerization.

To obtain functional evidence that SUMO chains activate RNF4 by induced dimerization of the RING domains, we employed a complementation strategy where two mutants of RNF4 that are each inactive as homodimers can display activity only if they can form heterodimers. Y193H RNF4 is inactive as it fails to interact with the ubiquitin component of the ubiquitin-loaded E2, whereas M140A+R181A RNF4 is inactive as it cannot bind the E2. If a heterodimer forms, the E2 component of the E2∼ubiquitin thioester binds to the Y193H subunit, while the ubiquitin component of the E2∼ubiquitin thioester engages Tyr193 of the M140A+R181A subunit, recreating a functional E3 ligase (Plechanovová et al., 2011, 2012). We therefore tested RNF4 autoubiquitination and lysine discharge activities of Y193H RNF4, M140A+R181A RNF4, or a mix of the two in the presence and absence of SUMO chains. In the absence of SUMO chains, neither Y193H, M140A+R181A, nor the mix of the two displays activity in either of the assays. In the presence of SUMO chains, M140A+R181A is inactive, Y193H has low activity, but the mix of the two mutants has robust E3 ligase activity in both assays (Figures 5C–5E). A similar assay in vivo also indicated that intramolecular complementation between RNF4 mutants was evident when SUMO chains were increased by ablation of SENP6 expression (Figure 5F). Thus SUMO chains induce the formation of functional dimers of RNF4.

### Quantitative Analysis of RNF4 Dimerization

**Figure 6:**
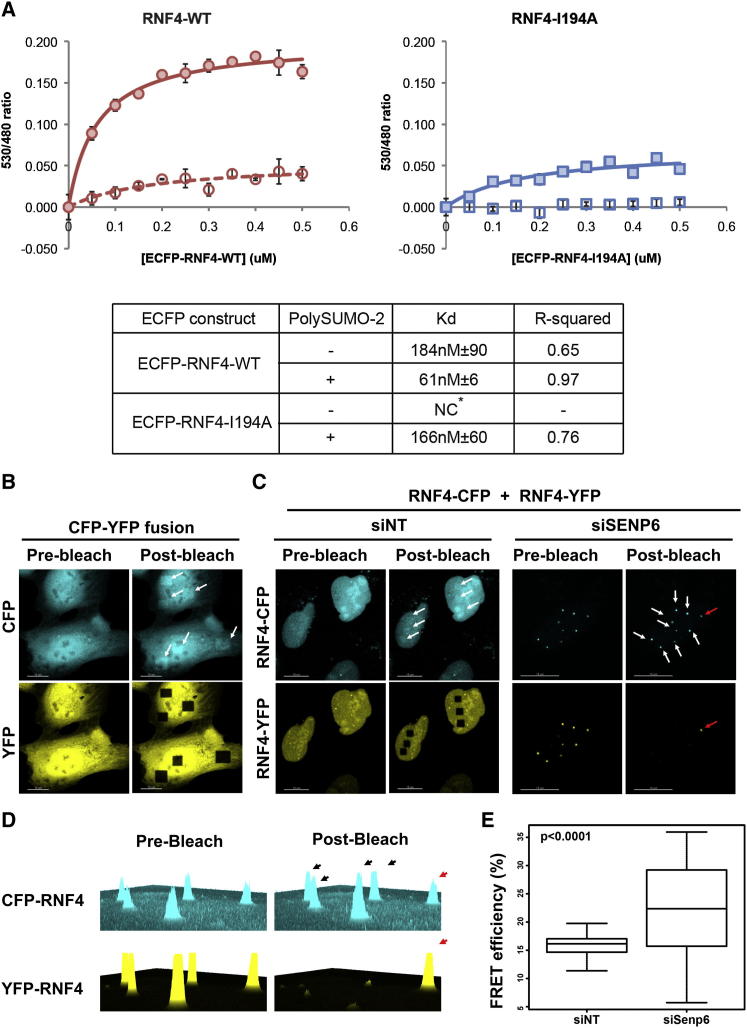
Dimerization of RNF4 in the Presence of SUMO Chains (A) In vitro FRET measurements with YFP-RNF4-WT or YFP-RNF4-I194A fixed at 0.5 μM and ECFP-RNF4-WT or ECFP-RNF4-I194A increased in 0.05 μM increments from 0 to 0.5 uM in the presence or absence of poly-SUMO-2. All reactions were made in triplicate. Average FRET signals and one standard deviation were calculated for each concentration point. To derive apparent K_d_ values, data were fit by nonlinear regression to a single binding site model (Graphpad Prism v4.0c). Data are presented as mean ± SD. (B) FRET acceptor photobleaching. CFP-YFP fusions were transfected in U2OS cells as positive control. (C) U2OS cells transfected either with siNT or siSENP6 and further with a RNF4-CFP and RNF4-YFP mix. (D) 2.5D intensity histogram of RNF4-CFP and RNF4-YFP signal pre- and postbleaching in an siSENP6 ablated cell. White and black arrows indicate bleached foci and a red arrow a nonbleach control foci. (E) Quantification of FRET efficiency (%) of acceptor photobleached regions in siNT and siSENP6 transfected cells, p < 0.0001 Student’s two-tailed t test. Data are presented as mean ± SD.

To quantitatively study the effect of SUMO-2 chains on the dimerization of RNF4, an in vitro Förster resonance energy transfer (FRET) assay was devised. Recombinant forms of RNF4 were expressed with N-terminal fusions of ECFP or YFP. When in close proximity, emission from ECFP can transfer between the fluorophores, causing emission from YFP (Martin et al., 2008). This assay was used to monitor polySUMO-2 effects upon dimerization of WT RNF4 and dimerization-deficient mutant I194A (Figure 6A). In the absence of polySUMO-2, dimerization of the wild-type protein was evident, whereas RNF4-I194A showed no evidence of dimerization (Figure 6A, left and right). Although the WT and mutant forms both generated FRET signals in the presence of polySUMO-2, the signal was substantially greater in the wild-type protein when compared to the dimerization-deficient mutant. Evidently, in absence of the ability to homodimerize, polySUMO-2 still causes RNF4 I194A to localize in close enough proximity to elicit a FRET signal. Thus these data show that SUMO polymers can recruit RNF4 in absence of dimerization activity, but also effectively increase affinity between wild-type RNF4 monomers. Fitting these data to a single binding site model revealed WT RNF4 to have a dimerization Kd of around 180 nM in absence of polySUMO-2, while in its presence the apparent Kd is reduced to around 60 nM (Figure 6A, lower panel). While this confirms tighter binding between RNF4 monomers, this figure is likely to reflect two events, namely the recruitment to polySUMO-2 chains either independent of, or dependent upon, homodimerization. Consistent with this, the nondimerizing mutant has an apparent Kd of ∼170 nM in the presence of polySUMO-2, but the maximum FRET signal is weaker than for the WT (Figure 6A, left and right). This indicates that WT and mutant proteins are not associating with one another in the same way. Thus, the higher FRET signal for WT RNF4 is indicative of the formation of closely associated active dimers bound to polySUMO-2, while the mutant is simply interacting with the SUMO polymer in a stochastic, monomeric way. In summary, these data show that SUMO polymers can recruit RNF4 in absence of dimerization activity, but also effectively increase affinity between wild-type RNF4 monomers.

To test the effect of increased SUMO chain concentration on RNF4 dimerization in vivo, SENP6 expression was ablated and RNF4 dimerization was monitored in an acceptor photobleaching FRET experiment. U2OS cells transfected with either siNT or siSENP6 siRNA were further transfected with RNF4-CFP and RNF4-YFP. At 24 hr posttransfection RNF4 fusions were localized at the nucleoplasm in siNT-transfected cells, while in the siSENP6-transfected cells RNF4 fusion proteins accumulated in SUMO-containing foci. In siNT-transfected cells YFP was photobleached in the nucleoplasm, while in siSENP6-treated cells YFP was photobleached in SUMO-containing foci, leaving at least one foci unbleached as negative control. YFP photobleaching of nuclear foci in siSENP6-treated cells led to an increase in fluorescence from the CFP donor, indicating association of RNF4 monomers to form dimers (Figures 6B–6D). Quantitation of this data indicated a highly significant (p < 0.0001) increase in FRET efficiency after SENP6 knockdown compared to siNT-transfected cells as well as a larger distribution of the data (Figure 6E), suggesting variability in the structure of the foci.

### Chemically Induced Dimerization of RNF4 Activates E3 Ligase Activity

**Figure 7:**
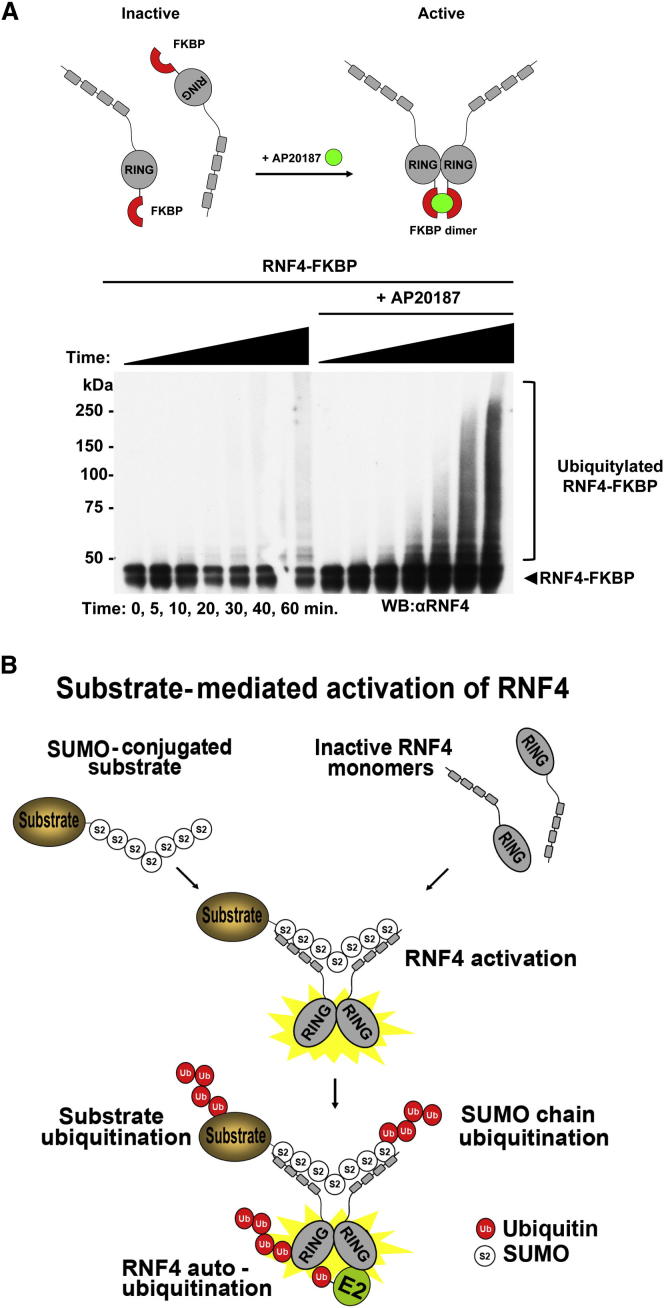
Chemical Induction of RNF4 Dimerization In Vitro (A) RNF4-FKBP in vitro ubiquitination reactions were performed either in the absence or presence of 1 μM AP20187. Autoubiquitination of RNF4-FKBP was analyzed by western blotting with an antibody against RNF4. (B) Model of RNF4 activation by polySUMO chains.

To demonstrate that SUMO chains function to bring RNF4 monomers into close enough proximity to induce dimerization, we sought to induce this increased proximity in the absence of SUMO chains. Thus we generated a recombinant version of RNF4 fused to FK506 binding protein (FKBP). FKBP is monomeric in solution but dimerizes with subnanomolar affinity in the presence of the homobifunctional molecule AP20187 (Spencer et al., 1993). RNF4-FKBP was therefore tested for in vitro autoubiquitination activity in the presence and absence of AP20187. In the absence of AP20187 only a low level of ubiquitination activity was observed, whereas in its presence robust autoubiquitination activity was evident (Figure 7A). Thus artificial dimerization of RNF4 in the absence of SUMO chains suggests that SUMO chains function by inducing the dimerization of RNF4.

## Discussion

The data presented here indicate that the STUbL RNF4 is predominantly monomeric and inactive in the absence of its polySUMO substrate. However, cellular accumulation of SUMO chains serves to recruit monomeric RNF4, creating a locally high concentration that allows the RING dimerization and activation of ubiquitination activity (Figure 7B). However, an alternative model for the action of chains is that they induce a conformational change in the RING domain of RNF4 that facilitates dimerization. It is difficult to definitively distinguish between these two possibilities. However, the evidence we have obtained suggests that there is not a unique requirement for SUMO chains to induce a conformation required for activity. Thus we previously demonstrated (Plechanovová et al., 2011) that the RING alone is active as an E3 ligase when at a concentration high enough for dimers to form. Here in Figure 7A we demonstrate that chemically induced dimerization can activate RNF4 E3 ligase activity in the absence of SUMO chains. In addition, an RNF4-RING fusion is fully active in the absence of SUMO chains. Based on our FRET-based dimerization assays, we estimate that the Kd for dimerization of RNF4, in the absence of SUMO chains, is about 200 nM. However, our estimate of the intracellular concentration of RNF4 based on quantitative western blotting is about 100 nM (Figure S4), which is well below the Kd for dimerization and consistent with our contention that RNF4 is predominantly monomeric in vivo unless SUMO chains are present.

Under normal growth conditions SUMO chains are maintained at low levels, and it is likely that this is a direct consequence of the deconjugating activity of the SUMO chain-specific proteases SENP6 (Hattersley et al., 2011; Mukhopadhyay et al., 2010) and SENP7 (Shen et al., 2009), as ablation of their expression leads to the accumulation of SUMO chains. During stress responses such as DNA damage, arsenic treatment, or heat shock, SUMO chains accumulate, and they would be predicted to activate RNF4. However, depending on the type of stress, this activation might be highly localized within the cell or on a more global scale. In the case of arsenic trioxide, SUMO chains form on PML protein present in nuclear bodies, and RNF4 is rapidly recruited to these sites in a SIM-dependent, but RING-independent, fashion (Geoffroy et al., 2010). Similarly, DNA damage results in the accumulation of SUMO-2/3 chains at sites of DNA damage, again resulting in the recruitment of RNF4 to these localized nuclear subdomains (Galanty et al., 2012; Guzzo et al., 2012; Vyas et al., 2013; Yin et al., 2012). In contrast, heat stress or proteasome inhibition results in a global increase of SUMO chains (Golebiowski et al., 2009; Tatham et al., 2011). After treatment with DNA damaging agents or arsenic trioxide, SUMO chains accumulate, but there is no evidence for a global degradation of RNF4. This is probably because the generation of SUMO chains and thus activation of RNF4 is limited by being transient and confined to distinct subnuclear localizations (Geoffroy and Hay, 2009; Yin et al., 2012). Although heat shock leads to a large increase in SUMO chains, this is again transient and does not appear to induce RNF4 degradation. In contrast, ablation of SENP6 expression leads to a long-term and global increase in the level of SUMO chains that ultimately induces RNF4 degradation.

In addition to RNF4 (Liew et al., 2010; Plechanovová et al., 2011), many RING type ubiquitin ligases including cIAP (Mace et al., 2008), TRAF6 (Yin et al., 2009), Mdm2-MdmX (Linke et al., 2008), BRCA1-BARD1 (Brzovic et al., 2001), and RING1b-Bmi1 (Buchwald et al., 2006) must form homo- or heterodimers to display ubiquitin ligase activity, and mutations that disrupt the dimer interface are defective for ubiquitin ligase activity (Budhidarmo et al., 2012). Recent studies have revealed that RING dimerization is required to recruit the ubiquitin-loaded E2 to the E3 and fold the ubiquitin back onto the E2 with one protomer of the E3 binding the E2, while both protomers of the E3 interact with ubiquitin (Plechanovová et al., 2012; Dou et al., 2012). These multiple interactions of the E3 with E2 and ubiquitin explain why RING dimerization is required for ubiquitin ligase activity. Given the critical role of dimerization in E3 ligase activity, it is not surprising that mechanisms have evolved to regulate this step. An excellent example of this type of regulation is evident with the Inhibitor of Apoptosis proteins (IAPs). The ligand for cIAP1 is the N-terminal tetra peptide (AVPI) of Second Mitochondrial Activator of Caspases (SMAC), and in the absence of this ligand cIAP1 adopts a compact, monomeric conformation in which the RING domain is occluded and the protein does not have ubiquitin ligase activity. Engagement of the antagonist ligand by the Baculoviral IAP Repeat (BIR) domain of cIAP1 induces a conformational change in the cIAP1 protein that releases the RING domain, facilitating dimerization and allowing the E3 ligase to assume its dimeric active state (Dueber et al., 2011; Feltham et al., 2011). SMAC mimetics, which are undergoing clinical trials as anticancer agents, function by inducing cIAP1 dimerization, autoubiquitination, and proteasomal degradation (Feltham et al., 2011; Dueber et al., 2011) in a manner that is reminiscent of the substrate-induced degradation of RNF4 reported here. Another potential example of this type of regulation is illustrated by the RNF146/iduna E3 ligase that targets proteins modified by polyADP ribose (PAR) for ubiquitination and proteasomal degradation. Although it is not known if PAR induces RING dimerization, it has been reported that addition of PAR to in vitro reactions containing RNF146/iduna dramatically stimulates both autoubiquitination and ubiquitination of PAR-modified substrates (Kang et al., 2011; Zhang et al., 2011).

There are many examples where proteins are converted into substrates for E3 ligases by posttranslational modifications such as phosphorylation or E3 ligases are activated by phosphorylation (de Bie and Ciechanover, 2011) or NEDD8 modification (Deshaies and Joazeiro, 2009). E3 ligases can also be activated by the binding of small molecules, such as auxin plant hormones (Calderon-Villalobos et al., 2010); however, an interesting aspect of the induction of RNF4 activity by SUMO chains is that the binding of the chain across the dimer means that the E3 ligase is not activated until the chain reaches a certain length. SUMO dimers, for instance, do not induce activation. Thus an attractive regulatory aspect of RNF4 is that its E3 ligase activation can be spatially and temporally restricted by the growth of SUMO chains.

## Experimental Procedures

### Antibodies

Chicken anti-RNF4 (Yin et al., 2012), sheep anti-SENP6 (Hattersley et al., 2011), sheep anti-SUMO-1 and sheep anti-SUMO-2 (Tatham et al., 2008), chicken anti-PML (Geoffroy et al., 2010), and sheep anti-UBC9 were prepared in house, antigen affinity purified; mouse anti-β-tubulin was from Sigma-Aldrish T0198, and mouse anti-GFP from Roche.

### siRNA

siRNA smart pools (Dhamacom) were reversely transfected using Lipofectamine RNAimax according to manufacturers’ instructions at a final concentration of 20 nM. Oligos were as follows: NT pool siNT (D-001810-10), siRNF4 smart pool (L-006557-00), siSENP6 (L-006044-00), siUBC9 (L-004910-00), siSENP6 oligo-2 (J-006044-06), siRNF4 non-ORF (5′ UTR 256 sense, GGGCAUGAAAGGUUGAGAAUU).

### Immunofluorescence and Microscopy

Cells imaging was performed by using wide-field deconvolutive microscopy, OMX-structured illumination microscopy (Hattersley et al., 2011; Schermelleh et al., 2008) and high-content imaging using an InCell2000 instrument as described in Supplemental Information.

### Disulfide Crosslink of RNF4

Based on a structure analysis of RNF4, we engineered a mutant able to form a disulfide dimer when dimerized. Complete protocol is detailed in Supplemental Information.

Further experimental details can be found in Supplemental Information.

## Supplemental Information

Document S1. Figures S1–S4, Table S1, and Supplemental Experimental Procedures

Movie S1. YFP-SUMO-2 Localization Recorded by Time Lapse HeLa YFP-SUMO-2 cells were transfected with siSENP6 pool. Twenty-four hours after transfection, YFP-SUMO-2 localization was recorded by time-lapse microscopy for 36 hr. Scale bars represent 20 μm. Right-bottom value indicates hours after transfection; see also Figure 2C.

Movie S2. RNF4-YFP Localization Recorded by Time Lapse HeLa RNF4-YFP cells were reverse transfected with siSENP6 in clear bottom microscopy chambers. Forty-eight hours after transfection, RNF4-YFP localization was recorded by time lapse microscopy for 24 hr. Scale bars represent 20 μm. Right-bottom value indicates hours after transfection; see also Figure 2H.
