# Supplementary material for: SUMO Chain-Induced Dimerization Activates RNF4
Source: Mol Cell. 2014 Mar 20;53(6):880–92. doi: 10.1016/j.molcel.2014.02.031 (PMC3991395; doi:10.1016/j.molcel.2014.02.031)
Supplement: Document S1. Figures S1–S4, Table S1, and Supplemental Experimental Procedures [file mmc1.pdf]

Molecular Cell, Volume *53*

Supplemental Information

SUMO Chain-Induced Dimerization Activates RNF4

Alejandro Rojas-Fernandez, Anna Plechanovová, Neil Hattersley, Ellis Jaffray,
Michael H. Tatham, and Ronald T. Hay

Figure S1

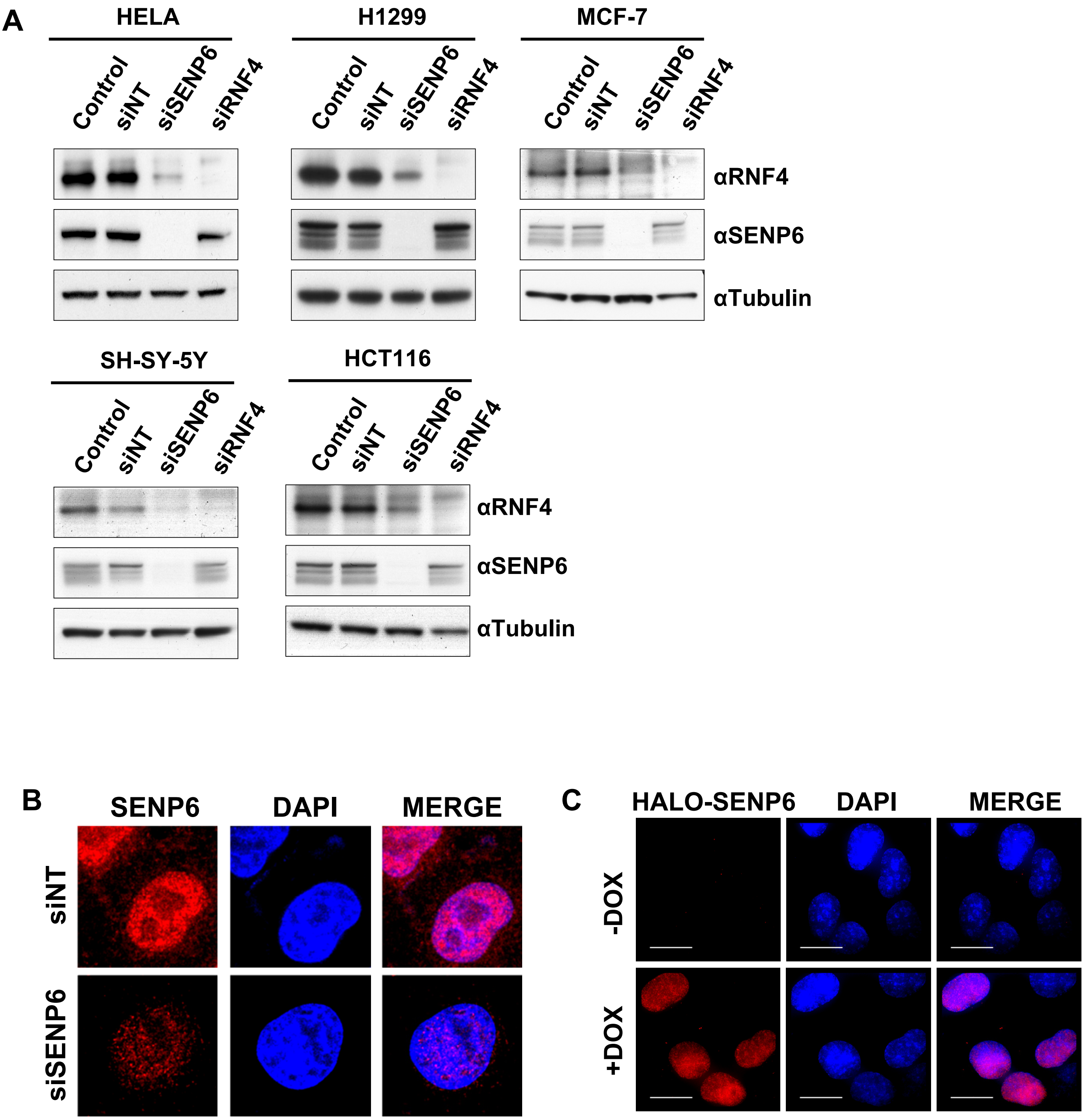
A
HELA
H1299
MCF-7
Control
siNT
siSENP6
siRNF4
αRNF4
αSENP6
αTubulin
SH-SY-5Y
HCT116
B
SENP6
DAPI
MERGE
siNT
siSENP6
C
HALO-SENP6
-DOX
+DOX

Figure S2

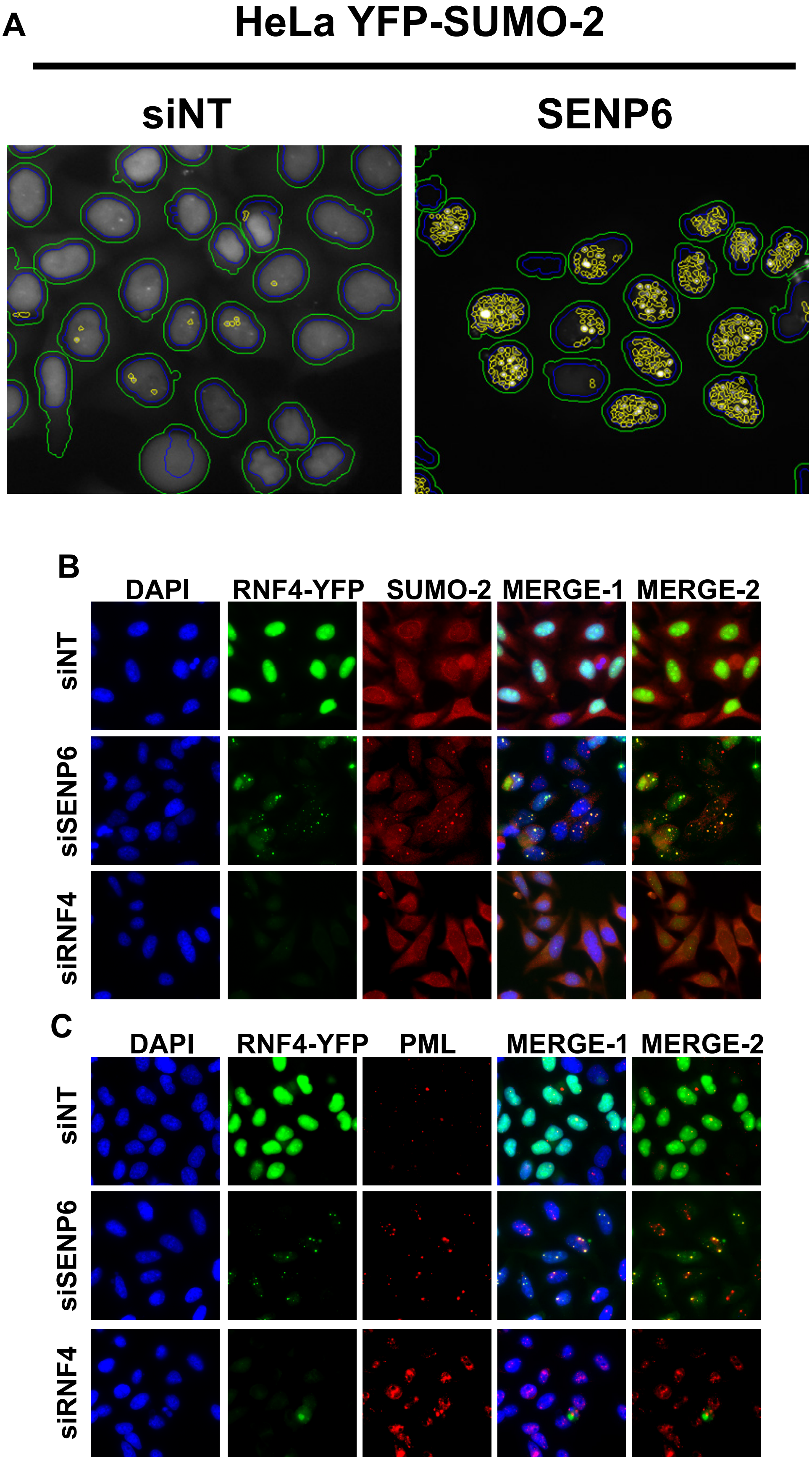
A
HeLa YFP-SUMO-2
siNT
SENP6
B
DAPI
RNF4-YFP
SUMO-2
MERGE-1
MERGE-2
siNT
siSENP6
siRNF4
C
DAPI
RNF4-YFP
PML
MERGE-1
MERGE-2
siNT
siSENP6
siRNF4

Figure S3

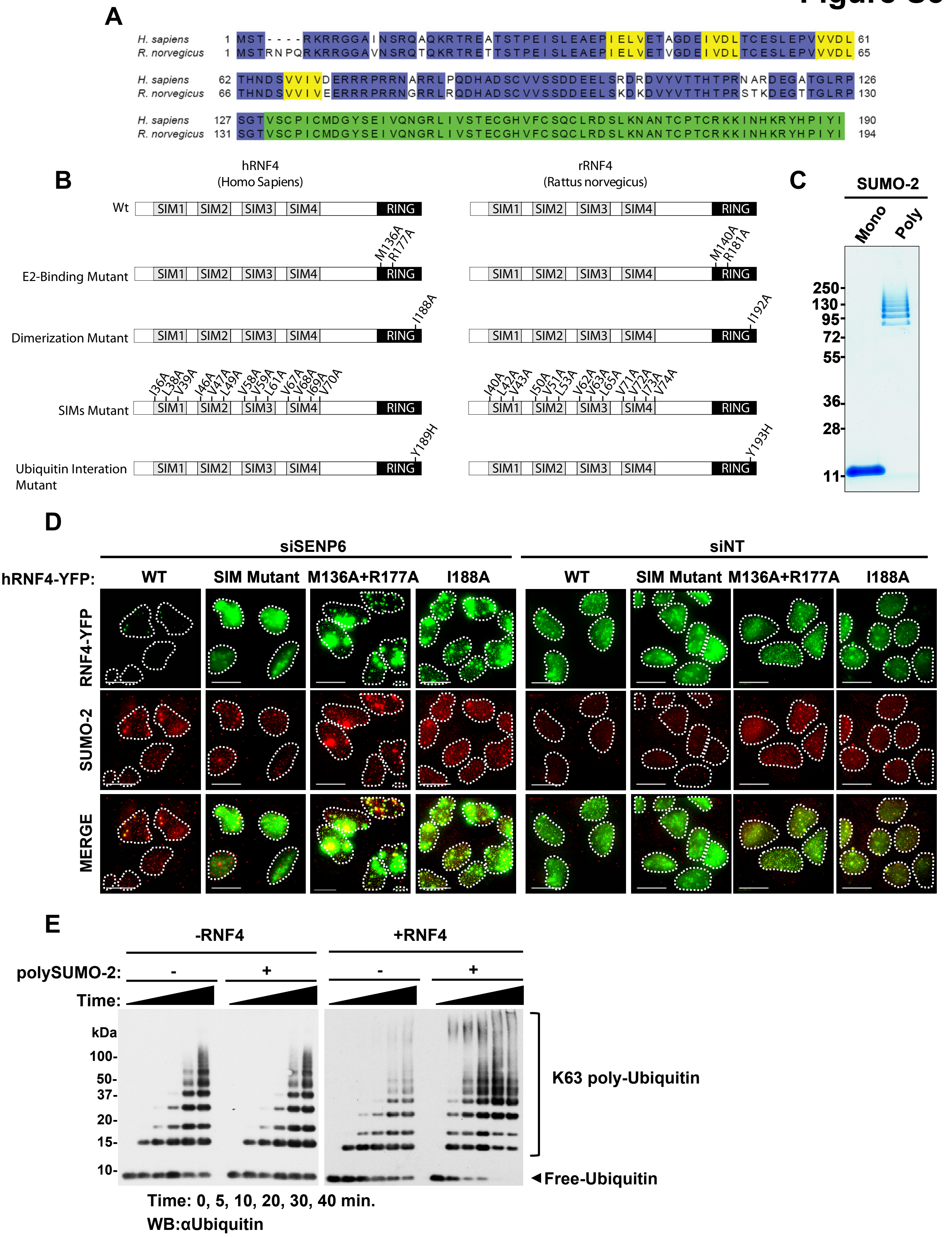
A
H. sapiens 1 MST----RKRRGGAINSRQAQKRTREATSTPEISLEAEPIELVETAGDEIVDLTCESLEPVVVDL 61
R. norvegicus 1 MSTRNPQRKRRGGAVNSRQTQKRTRETTSTPEISLEAEPIELVETVGDEIVDLTCESLEPVVVDL 65
H. sapiens 62 THNDSVVIVDERRRPRRNARRLPQDHADSCVVSSDDEELSRDRDVYVTTHTPRNARDEGATGLRP 126
R. norvegicus 66 THNDSVVIVEERRRPRRNGRRLRQDHADSCVVSSDDEELSKDKDVYVTTHTPRSTKDEGTTGLRP 130
H. sapiens 127 SGTVSCPICMDGYSEIVQNGRLIVSTECGHVFCSQCLRDSLKNANTCPTCRKKINHKRYHPIYI 190
R. norvegicus 131 SGTVSCPICMDGYSEIVQNGRLIVSTECGHVFCSQCLRDSLKNANTCPTCRKKINHKRYHPIYI 194
B
hRNF4 (Homo Sapiens)
rRNF4 (Rattus norvegicus)
Wt
E2-Binding Mutant
Dimerization Mutant
SIMs Mutant
Ubiquitin Interation Mutant
SIM1 SIM2 SIM3 SIM4 RING
M136A R177A
M140A R181A
I188A
I192A
I36A L38A V39A I46A V47A L49A V58A V59A L61A V67A V68A I69A V70A
I40A L42A V43A I50A V51A L53A V62A V63A L65A V71A V72A I73A V74A
Y189H
Y193H
C
SUMO-2
Mono Poly
250- 130- 95- 72- 55- 36- 28- 11-
D
siSENP6
siNT
hRNF4-YFP:
WT SIM Mutant M136A+R177A I188A
RNF4-YFP
SUMO-2
MERGE
E
-RNF4
+RNF4
polySUMO-2: - + - +
Time:
kDa
100- 50- 37- 20- 15- 10-
K63 poly-Ubiquitin
◄Free-Ubiquitin
Time: 0, 5, 10, 20, 30, 40 min.
WB:αUbiquitin

Figure S4

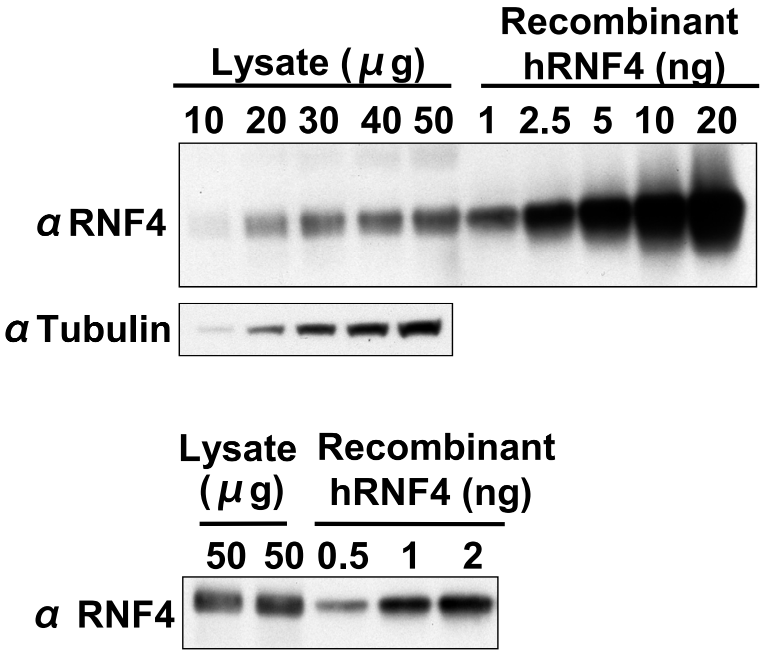
Lysate (μg)
Recombinant hRNF4 (ng)
10 20 30 40 50 1 2.5 5 10 20
αRNF4
αTubulin
Lysate (μg)
Recombinant hRNF4 (ng)
50 50 0.5 1 2
α RNF4

Table-S1

Oligo-1	M136A forward	GTCAGTTGTCCCATCTGCGCGGACGGATACTCAGAGATCGTG
Oligo-2	M136A reverse	CACGATCTCTGAGTATCCGTCCGCGCAGATGGGACAACTGAC
Oligo-3	R177A forward	CTAATACTTGCCCAACTTGTGCGAAAAAGATCAACCACAAAC
Oligo-4	R177A reverse	GTTTGTGGTTGATCTTTTTCGCACAAGTTGGGCAAGTATTAG
Oligo-5	I188A forward	CCACAAACGGTACCACCCCGCTTATATATGA
Oligo-6	I188A reverse	TCATATATAAGCGGGGTGGTACCGTTTGTGG
Oligo-7	SIM1:I36A, L38A, V39A forward	GATCTCCTTGGAAGCAGAACCCGCAGAAGCCGCGGAAACTGCTGGAGATG
Oligo-8	SIM1:I36A, L38A, V39A reverse	CATCTCCAGCAGTTTCCGCGGCTTCTGCGGGTTCTGCTTCCAAGGAGATC
Oligo-9	SIM2: I46A, V47A, L49A forward	GAAACTGCTGGAGATGAAGCTGCGGACGCCACTTGTGAATCTTTAGAG
Oligo-10	SIM2: I46A, V47A, L49A reverse	CTCTAAAGATTCACAAGTGGCGTCCGCAGCTTCATCTCCAGCAGTTTC
Oligo-11	SIM3 V58A, V59A, L61A forward	CTTGTGAATCTTTAGAGCCTGTGGCGGCTGATGCGACTCACAATGACTC
Oligo-12	SIM3 V58A, V59A, L61A reverse	GAGTCATTGTGAGTCGCATCAGCCGCCACAGGCTCTAAAGATTCACAAG
Oligo-13	SIM4 V67A, V68A, I69A, V70A forward	GCGACTCACAATGACTCTGCTGCGGCTGCTGACGAAAGAAGAAGACCAAGG
Oligo-14	SIM4 V67A, V68A, I69A, V70A reverse	CCTTGGTCTTCTTCTTTCGTCAGCAGCCGCAGCAGAGTCATTGTGAGTCGC
Oligo-15	Senp6 siRNA resistant-2 forward	GTGAAGGAGATACAGATAAAGATGGTACGAACTTATTGAGCGTGGATGAAGATGAGGATTCTGAAACCTC
Oligo-16	Senp6 siRNA resistant-2 reverse	GAGGTTTCAGAATCCTCATCTTCATCCACGCTCAATAAGTTCGTACCATCTTTATCTGTATCTCCTTCAC
Oligo-17	5’ hRNF4 BamH1	GGTGCCGGATCCATGAGTACAAGAAAGCGTCGTGG
Oligo-18	3’ hRNF4 Stop XhoI	CTGTTCTCGAGTCATATATAAATGGGGTGGTACC
Oligo-19	5’ Y189H RNF4	CCACAAACGGTACCACCCCATTCATATATGACTCGAGATGCATACGCGTGC
Oligo-20	3’ Y189H RNF4	GCACGCGTATGCATCTCGAGTCATATATGAATGGGGTGGTACCGTTTGTGG
Oligo-21	3’ hRNF4 BamH1 no stop	CCGCGGATCCTATATAAATGGGGTGGTACC
Oligo-22	5’ hRNF4 Ring BamH1	CCGCGGATCCCATACTCCCAGAAACGCCAGG
Oligo-23	3’ hRNF4 Ring XhoI Stop	CTGTTCTCGAGTCATATATAAATGGGGTGGTACC
Oligo-24	5’ hRNF4 Nco1	GGTGCCATGGGGATGAGTACAAGAAAGCGTCGTGG
Oligo-25	3’ hRNF4 EcoR1 no stop	CCGCGAATTCTATATAAATGGGGTGGTACC
Oligo-26	5’ EcoR1 FKBP	CATTGAATTCATGGCTTCTAGAGGAGTGCAGGTG
Oligo-27	3’ XhoI FKBP	CATTCTCGAGTGCGTAGTCTGGTACGTCGTACGG
Oligo-28	5’ RNF4+GLY195+Cys196	CCATCCCATTTATATAGGGTGCTGATAAAAGCTTGGCACTGGCC
Oligo-29	3’ RNF4+GLY195+Cys196	GGCCAGTGCCAAGCTTTTATCAGCACCCTATATAAATGGATGG

Supplemental Figure Legends

Figure S1. Ablation of SENP6 reduces RNF4 levels in multiple cell lines (Related to Figure 1). A) HeLa, H1299, MCF-7, SH-SY-5Y and HCT116 cells untransfected (control) or transfected with siRNA non-targeting (siNT), siSENP6 or siRNF4 were incubated for 72h. Protein levels were analyzed by western blotting using specific antibodies against RNF4, SENP6 and Tubulin. **B)** HeLa cells were treated with either non-targeting or SenP6 specific siRNA, fixed with formaldehyde, permeabilised with Triton X100 and labeled with SenP6 antibodies (Red) and DAPI to visualise DNA (Blue). Images are presented as maximal intensity projections.

C) Microscope images of U2OS Flp T-Rex HALO-SENP6 cells. Cells were untreated (control) or induced with doxycyclin for 48h, fixed and stained with HALO-Tag TMR Ligand (red) and DAPI (blue). Scale bars represent 5μm.

Figure S2. High content imaging and RNF4 localisation (Related to Figure 2). A) Quantification of YFP-SUMO-2 nuclear bodies was performed by using high content imaging in a InCell2000 automatic microscope. The InCell200 software recognised the nucleus based on DAPI staining (blue lines) and nuclear bodies within the nucleus (yellow lines). Example of YFP-SUMO-2 HELA cells transfected either with siRNA siNT or siSENP6 and incubated for 72h before analysis. **B)** HeLa RNF4-YFP (green) cells were transfected with siRNA pools siNT or siSENP6 and incubated for 72h and further immunostained with a SUMO-2 antibody (red). **C)** HeLa RNF4-YFP (green) cells were transfected with siRNA pools siNT or siSENP6 and incubated for 72h and further immunostained with a PML antibody (red). Scale bars represent 5μm.

Figure S3. RNF4 mutants (Related to Figure 4). A) *Homo sapiens* and *Rattus norvegicus* RNF4 aligment. **B)** Diagram of RNF4 mutants of human and rat RNF4 use in this study. **C)** Bacterial purified monomeric SUMO-2 and long polySUMO-2 chains (>4 mers), proteins are visualized by coomassie staining. **D)** Stable U2OS cell lines encoding either human RNF4 fused to YFP, a SIM mutant of RNF4-YFP, an E2 binding deficient mutant of RNF4-YFP (M136A, R177A) or a dimerisation defective version of RNF4-YFP (I188A) were transfected with an siRNA oligo targeting only endogenous RNF4 (siRNF4 non-ORF4). After 24h incubation, cells were transfected again either with siRNAs siNT or an siRNA to SENP6 and incubated for additional 72h. RNF4-YFP wt and mutants localization (green) was analysed together with endogenous SUMO immunostaining (red). Images of SENP6 depleted cells and non-targeting transfected were obtain under the same conditions and intensity was normalized to the respective siNT control of each RNF4-YFP version. Scale bars represent 5µm (dashed lines show the localization of the nucleus determined by DAPI staining). E) Formation of unanchored K63 chains by Uev2/Ubc13. Uev2/Ubc13 generates unanchored K63 chains in the absence of RNF4 and this activity is not influenced by the presence of SUMO chains (left panel). Addition of RNF4 modestly increased K63 chains synthesis, but in the presence RNF4 and SUMO chains long K63 polymers accumulate and free ubiquitin is depleted (right panel). Unanchored K63 Ubiquitin chains were determined by Westen blot using an anti-Ubiquitin antibody.

Figure S4. Quantification of RNF4 levels in HELA cells (Related to Figure 6). Western blotting using a specific RNF4 antibody was performed loading 50µg of total cell extract and quantified by using recombinant hRNF4 as a standard.

Table S1. List of DNA oligonucleotides (Related to Experimental Procedures)

List of DNA oligonucleotides use in this study (life technologies) for PCR amplification and mutagenesis PCR.

Movie 1. YFP-SUMO-2 localization recorded by time-lapse (Related to Figure 2)

HeLa YFP-SUMO-2 cells were transfected with siSENP6 pool. 24h after transfection YFP-SUMO-2 localization was recorded by time-lapse microscopy for 36h. Scale bars represent 20μm. Right-bottom value indicates hours after transfection, see also Figure 2C.

Movie 2. RNF4-YFP localization recorded by time lapse (Related to Figure 2)

HeLa RNF4-YFP cells were reverse transfected with siSENP6 in clear bottom microscopy chambers, 48 hr after transfection RNF4-YFP localization was recorded by time lapse microscopy for 24h. Scale bars represent 20μm. Right-bottom value indicates hours after transfection, see also Figure 2H.

Extended Experimental Procedures

Cloning and plasmid

The full-length human pEF-BOS-hRNF4-YFP was described in previous publications (Mizushima and Nagata, 1990; Yin et al., 2012). RNF4-YFP mutants were generated by mutagenesis PCR using KOD Hot Start DNA polymerase (Novagen). The following oligos were used to generate an hRNF4-YFP E2 binding impaired M136A + R177A, for the M136A mutant oligo-1 (forward) and oligo-2 (reverse), for R177A mutant oligo-3 (forward) and oligo-4 (reverse). hRNF4-YFP dimerisation mutant I188A was generated with oligo-5 (forward) and oligo-6 (reverse). hRNF4-YFP SIM mutant was generated in 4 consecutive steps of mutagenesis PCR, i) SIM1 mutagenesis I36A, L38A, V39A using hRNF4-YFP wt as template with primers oligo-7 (forward) and oligo-8 (reverse) ii) SIM1+SIM2 mutagenesis I46A, V47A, L49A using hRNF4-YFP SIM1 mutant as template with primers oligo-9 (forward) and oligo-10 (reverse) iii) SIM1+SIM2+SIM3 mutagenesis V58A, V59A, L61A using hRNF4-YFP SIM1+SIM2 mutant as template with primers oligo-11 forward and oligo-12 reverse: iv) SIM1+SIM2+SIM3+SIM4 mutagenesis V67A, V68A, I69A, V70A using hRNF4-YFP SIM1+SIM2+SIM3 mutant as template with primers oligo-13 (forward) and oligo14 (reverse).

To generate pcDNA5 FRT/TO-HALO-tev-SENP6 siRNA resistant against SENP6 oligo-2 (J-006044-06) first we cloned SENP6 (NM_015571.2) using restriction enzymes BamHI and NotI into pcDNA5 FRT/TO-HALO-tev vector (DSTT, MRC-PUU Dundee University). Further we mutated the recognition site of the oligo-2 sequences by mutagenesis PCR using the following primers oligo-15 forward and oligo-16

reverse. pEF-BOS-rRNF4 CFP and pEF-BOS-rRNF4 YFP as previously descried (Geoffroy et al., 2010).

Flag tagged hRNF4 variants were cloned between sites BglII and XhoI of the pFires-Puro vector DSTT University of Dundee. hRNF4 wt using primers Oligo-17 BamH1 and Oligo-18 XhoI, hRNF4 Y189H using was generated by mutagenesis PCR using primers oligo-19 and oligo-20. Flag tagged hRNF4-RING was clone in a triple fragment ligation full hRNF4 was amplify by using primers oligo-17 BamH1 and oligo-21 no stop BamH1, hRNF4 RING domain was amplified with oligo-22 BamH1 and Oligo-23 XhoI primers and further digestion hRNF4 full and RING domain were ligated simultaneously into pFires-Puro Vector.

RNF4-C196 was cloned by mutagenesis PCR with oligo-28 and oligo-29 using the vector pLou3-RNF4 (rat) C55S C95S as template.

hRNF4-FKBP fusion was cloned into the bacterial expression vector pET30a, hRNF4 full length was amplified by PCR using oligo-24 NcoI and oligo-25 EcoR1 no stop FKBP domain was amplified by PCR from the vector pC4-Fv1E (ARGENT) using primers oligo-26 EcoR1 and oligo-27 XhoI. hRNF4 full length and FKBP domain were ligates together into pET30a vector digested with NcoI and XhoI enzymes.

All plasmids were checked carefully by sequencing the entire insert. Oligo sequences are provided in the supplementary Table-1.

High content transfection and imaging

µClear bottom 96 well plates (Greiner bio-one) were reverse transfected with Smartpool siRNAs using 6.000 cells per well. 72 hr after transfection cells were stained automatically in a fluid X dispenser (100 µL per step) using the following protocol: 2 washes with phosphate buffered saline (PBS), 10 minutes fixation in 3.7% Paraformaldehyde (Sigma), 1 wash with PBS, 10 minutes incubation with 0.2% Triton X100 (VWR) in PBS, 2 washes with PBS, 5 minutes incubation with 0.1 µg/ml DAPI (SIGMA), 2 washes with PBS. Finally 100 µL PBS were left in each well. Images were acquired and analysed using an InCell 2000 (GE automatic microscope). 6 randomized fields were acquired having ~ 600 cell each n > 2000 cells per condition.

Cell culture and transfection

HeLa and U2OS cells were maintained at 37°C and 5% CO_2 in DMEM supplemented wth 10% fetal calf serum (FCS), and penicillin/streptomycin at 100 U/ml. Cells stably expressing YFP-SUMO-2 were in addition supplemented with 250 µg/ml G418. Cells stably expressing RNF4-YFP wt, RNF4-YFP E2 binding impaired mutant M136A + R177A, RNF4 dimerisation mutant I188A and RNF4-YFP SIM mutant were selected and maintained in 15 µg/ml blasticidin, single cell clones were isolated from 15cm plates using cloning cylinder, glass (Sigma-Aldrich). U2OS Flp-in T-REX HALO-SENP6 siRNA resistant were maintained in DMEM +10%FBS supplemented with hygromycin (100 µg/ml), zeocin (100 µg/ml) and blasticidin (15 µg/ml) single cell clones were isolated from 15cm plates using cloning cylinder, glass (Sigma-Aldrich).

Plasmid transfection was performed using Lipofectamine 2000 (Invitrogen) according to manufacturer's instructions. The respective media were supplemented with Normocin (Invivogene).

Immunofluorescence and microscopes.

Cells were reverse transfected with siRNAs on 12mm coverslips prior treatments when indicated, fixed for 10 minutes in 3.7% paraformaldehyde/PBS at room temperature for 20 minutes, permeabilized in 0.2% Triton X-100/PBS for 5 minutes, blocked in 10%FBS in PBS for 1 hr. First antibodies diluted in 5%FBS in PBS was incubated 1 hr at room temperature. Further cell were washed 3 times with PBS and stained with secondary antibodies diluted in 5%FBS in PBS for 1hr at room temperature in the dark. Cells were washed one time and then incubated 5 minutes with 0.1 µg/ml DAPI. Finally, the samples were washed 3 times with PBS, 2 times with water and 1 time with absolute ethanol before mounting the samples on microscope slices with Vectashield (Vector H-1000).

Live-cell imaging was performed by seeding stably expressing YFP–SUMO-2 or RNF4-YFP cells into clear bottom chambers LabTek (Bloomington, IN) in Liebovitz CO_2 independent medium and imaged on a DeltaVision Spectris widefield microscope fitted with a 37°C environment chamber (Solent Scientifi c, Segensworth, United Kingdom). Structured illumination microscopy was performed as previously described (Hattersley et al., 2011; Schermelleh et al., 2008).

In cells FRET acceptor photo bleaching was performed in a M710 zeiss microscope using a 100% of 514nm laser 20 iterations for YFP bleaching.

Proteins and *in vitro* ubiquitylation assays

Expression and purification of human UbcH5a, *R. norvegicus* RNF4 and the RNF4-RING linear fusion (a linear fusion of full-length RNF4 and the RNF4 RING domain) was described previously (Plechanovova et al., 2011). Ubiquitin was obtained from Sigma (cat. no. U6253). SUMO-2 chains were prepared by incubating SUMO-2 (500 μM) with GST-tagged SAE1/SAE2 (0.26 μM) and His_6-tagged Ubc9 (16 μM) at 37°C for ~9 hours. Reaction buffer contained 50 mM Tris (pH 7.5), 3 mM ATP, 5 mM $MgCl_2$, and 5 mM DTT. Subsequently, SAE1/SAE2 and Ubc9 were removed by glutathione- and nickel-affinity chromatography, respectively. SUMO-2 chains were resolved by gel filtration chromatography on a HiLoad 16/60 Superdex 75 column (GE Healthcare).

In vitro RNF4 ubiquitination reactions contained 100 nM His_6-tagged human Ube1, 500 nM human UbcH5a, 20 μM ubiquitin, and *R. norvegicus* RNF4 (140 nM unless indicated otherwise in the figure legend). Reactions were performed either in the absence or presence of SUMO-2 or SUMO-2 chains, as stated in the figure legends. Samples were buffered with 50 mM Tris (pH 7.5), 150 mM NaCl, 0.5 mM TCEP, 0.1% (v/v) NP40, 3 mM ATP, 5 mM $MgCl_2$. Reactions were incubated at room temperature and stopped with SDS-PAGE loading buffer. “Zero” time point was taken before reactions were started by addition of ATP. Autoubiquitination of RNF4 was analysed by Western blotting with chicken anti-RNF4 antibody.

Disulfide crosslink of RNF4

Based on a structure analysis of RNF4 we engineered a mutant able to form a disulfide dimer when dimerised. RNF4-Two cysteins on RNF4 that are not involved in zinc coordination in the RING finger domain were mutated to serine to generate an

RNF4 C55S, C95S mutant. Next, one glycine was inserted at the C-terminus of the protein as linker (glycine 195) followed by a cysteine to form a disulfide bond when RNF4 dimerised (cysteine 196) RNF4 C196. Disulfide dimerisation was induced by depletion of DTT by buffer exchanges with micro spin columns 3KDa (Amicon, Millipore) and further incubation at room temperature in 50mM Tris-HCl, 50mM NaCl, pH7.5.

Cysteine residues in recombinant monomeric SUMO-2 and (>4) SUMO-2 chains were alkylated in 50mM idoacetamide for 20h at 4°C. DTT was added to a final concentration of 25mM and incubated for 30 minutes at room temperature. Samples were buffer exchanged on 3KDa micro spin columns (Amicon, Millipore) into 50mM Tris-HCl, 150mM NaCl, pH 7.5. Alkylated monomeric SUMO-2 or poly-SUMO-2 chains were added to RNF4 C196 as indicated.

Lysine discharge assays and measuring the formation of K63 unanchored chains.

Lysine discharge assays were carried out essentially as described previously (Plechanovova et al., 2011). Assays were carried out in triplicate and after SDS PAGE gels were stained with Sypro Ruby and quantified on a BIORAD ChemiDoc Imager. Assays measuring the formation of K63 unanchored chains by Uev2/Ubc13 in response to RNF4 were conducted as described (Tatham et al., 2013).

Calculation of apparent Kd values in vitro using FRET

YFP, YFP-RNF4-WT, YFP-RNF4-I194A, ECFP-RNF4-WT and ECFP-RNF4-I194A proteins were expressed and purified as described previously (Martin et al., 2008). In vitro FRET measurements were made on a BMG labtech Novostar platereader using

384 well clear bottom black sided plates (Corning type 3711). Measurements were made using excitation filter 405-20 with dual emission collection at 480-10 and 530-10. In each well reaction volumes of 40uL were used, buffered in 50mM Tris/HCl pH 7.5, 150mM NaCl, 0.5mM TCEP and 0.1% NP-40. YFP-RNF4-WT or YFP-RNF4-I194A were fixed at 0.5uM and ECFP-RNF4-WT or ECFP-RNF4-I194A concentration ranges from 0 to 0.5 uM were made in 0.05uM increments. Reactions containing poly-SUMO-2 contained 10uM polymer. Assays containing YFP in place of the YFP-RNF4 variants were also prepared as controls. All reactions were made in triplicate. Due to the very low concentrations of fluorescent proteins ratios of emissions at 530nm and 480nm were used in calculations to reduce improve sensitivity. FRET signals were extracted by subtraction of YFP control ratios from experimental data at each concentration point and then the data normalized to the ratio at 0uM ECFP-RNF4. Average FRET signals and one standard deviation were calculated for each concentration point. Data were fit by non-linear regression to a single binding site model (Graphpad Prism v4.0c).

Supplemental References

Geoffroy, M.C., Jaffray, E.G., Walker, K.J., and Hay, R.T. (2010). Arsenic-induced SUMO-dependent recruitment of RNF4 into PML nuclear bodies. Mol Biol Cell *21*, 4227-4239.

Hattersley, N., Shen, L., Jaffray, E.G., and Hay, R.T. (2011). The SUMO protease SENP6 is a direct regulator of PML nuclear bodies. Mol Biol Cell *22*, 78-90.

Martin, S.F., Tatham, M.H., Hay, R.T., and Samuel, I.D. (2008). Quantitative analysis of multi-protein interactions using FRET: application to the SUMO pathway. Protein Sci *17*, 777-784.

Mizushima, S., and Nagata, S. (1990). pEF-BOS, a powerful mammalian expression vector. Nucleic Acids Res *18*, 5322.

Plechanovova, A., Jaffray, E.G., McMahon, S.A., Johnson, K.A., Navratilova, I., Naismith, J.H., and Hay, R.T. (2011). Mechanism of ubiquitylation by dimeric RING ligase RNF4. Nat Struct Mol Biol *18*, 1052-1059.

Schermelleh, L., Carlton, P.M., Haase, S., Shao, L., Winoto, L., Kner, P., Burke, B., Cardoso, M.C., Agard, D.A., Gustafsson, M.G., *et al.* (2008). Subdiffraction multicolor imaging of the nuclear periphery with 3D structured illumination microscopy. Science *320*, 1332-1336.

Tatham, M.H., Plechanovova, A., Jaffray, E.G., Salmen, H., and Hay, R.T. (2013). Ube2W conjugates ubiquitin to alpha-amino groups of protein N-termini. Biochem J *453*, 137-145.

Yin, Y., Seifert, A., Chua, J.S., Maure, J.F., Golebiowski, F., and Hay, R.T. (2012). SUMO-targeted ubiquitin E3 ligase RNF4 is required for the response of human cells to DNA damage. Genes Dev *26*, 1196-1208.
