# Supplementary figures and images for: SUMO Chain-Induced Dimerization Activates RNF4
Source: Mol Cell. 2014 Mar 20;53(6):880–92. doi: 10.1016/j.molcel.2014.02.031 (PMC3991395; doi:10.1016/j.molcel.2014.02.031)

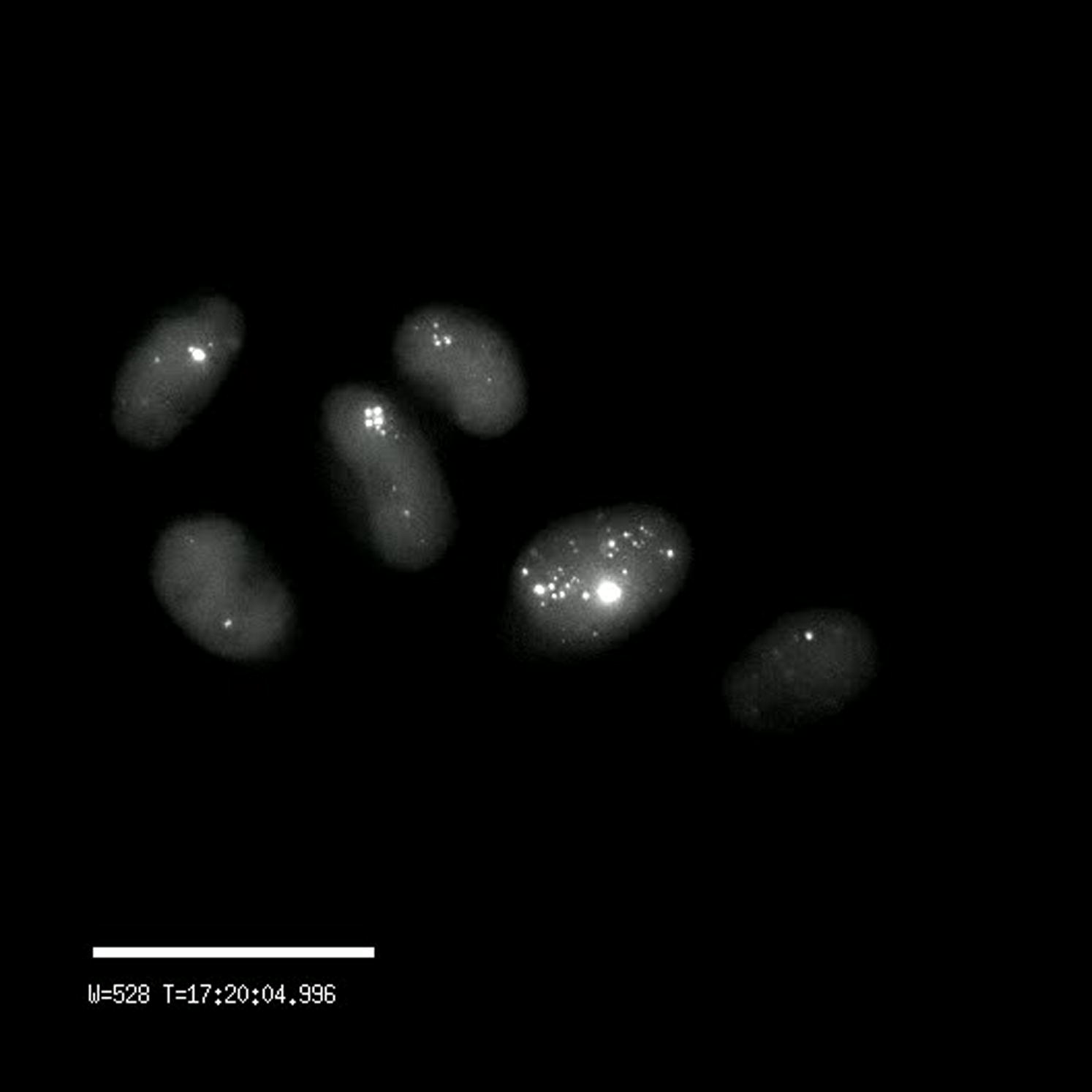

Supplement: Movie S1. YFP-SUMO-2 Localization Recorded by Time Lapse — HeLa YFP-SUMO-2 cells were transfected with siSENP6 pool. Twenty-four hours after transfection, YFP-SUMO-2 localization was recorded by time-lapse microscopy for 36 hr. Scale bars represent 20 μm. Right-bottom value indicates hours after transfection; see also Figure 2C. [file mmc2.jpg]

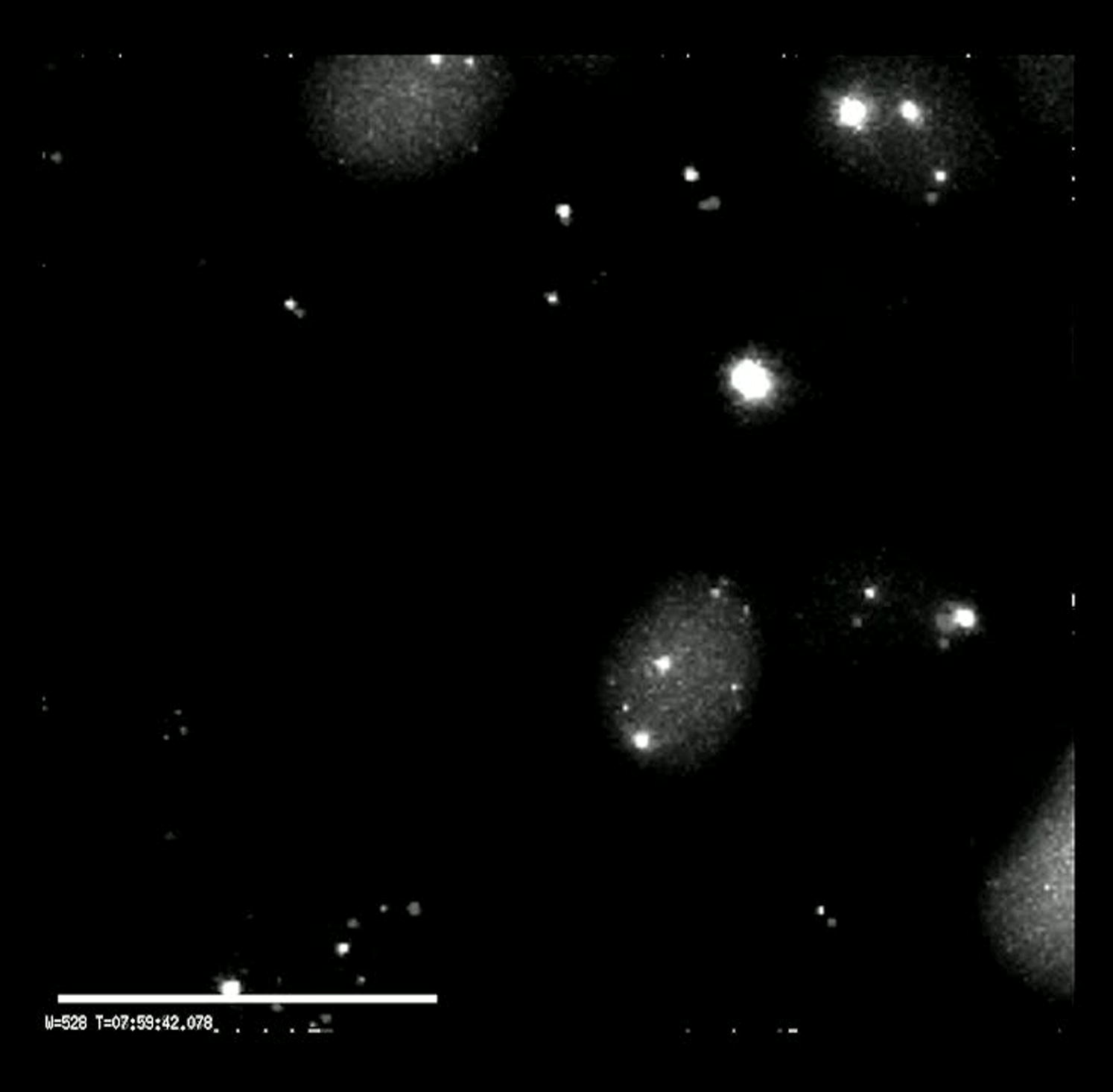

Supplement: Movie S2. RNF4-YFP Localization Recorded by Time Lapse — HeLa RNF4-YFP cells were reverse transfected with siSENP6 in clear bottom microscopy chambers. Forty-eight hours after transfection, RNF4-YFP localization was recorded by time lapse microscopy for 24 hr. Scale bars represent 20 μm. Right-bottom value indicates hours after transfection; see also Figure 2H. [file mmc3.jpg]
